# Correction: Unraveling Patterns of Site-to-Site Synonymous Rates Variation and Associated Gene Properties of Protein Domains and Families

**DOI:** 10.1371/journal.pone.0102721

**Published:** 2014-07-08

**Authors:** The PLOS ONE Staff

## Notice of Republication

This article was republished on June 18, 2014, to replace Figure 3, which was an accidental duplication of Figure 2. Please download this article again to view the correct version. The originally published, uncorrected article and the republished, corrected article are provided here for reference.

## Supporting Information

File S1 Originally published, uncorrected article.
(PDF)

File S2 Republished, corrected article.
(PDF)
