# Supplementary figures and images for: Unraveling Patterns of Site-to-Site Synonymous Rates Variation and Associated Gene Properties of Protein Domains and Families
Source: PLoS One. 2014 Jun 4;9(6):e95034. doi: 10.1371/journal.pone.0095034 (PMC4045579; doi:10.1371/journal.pone.0095034)

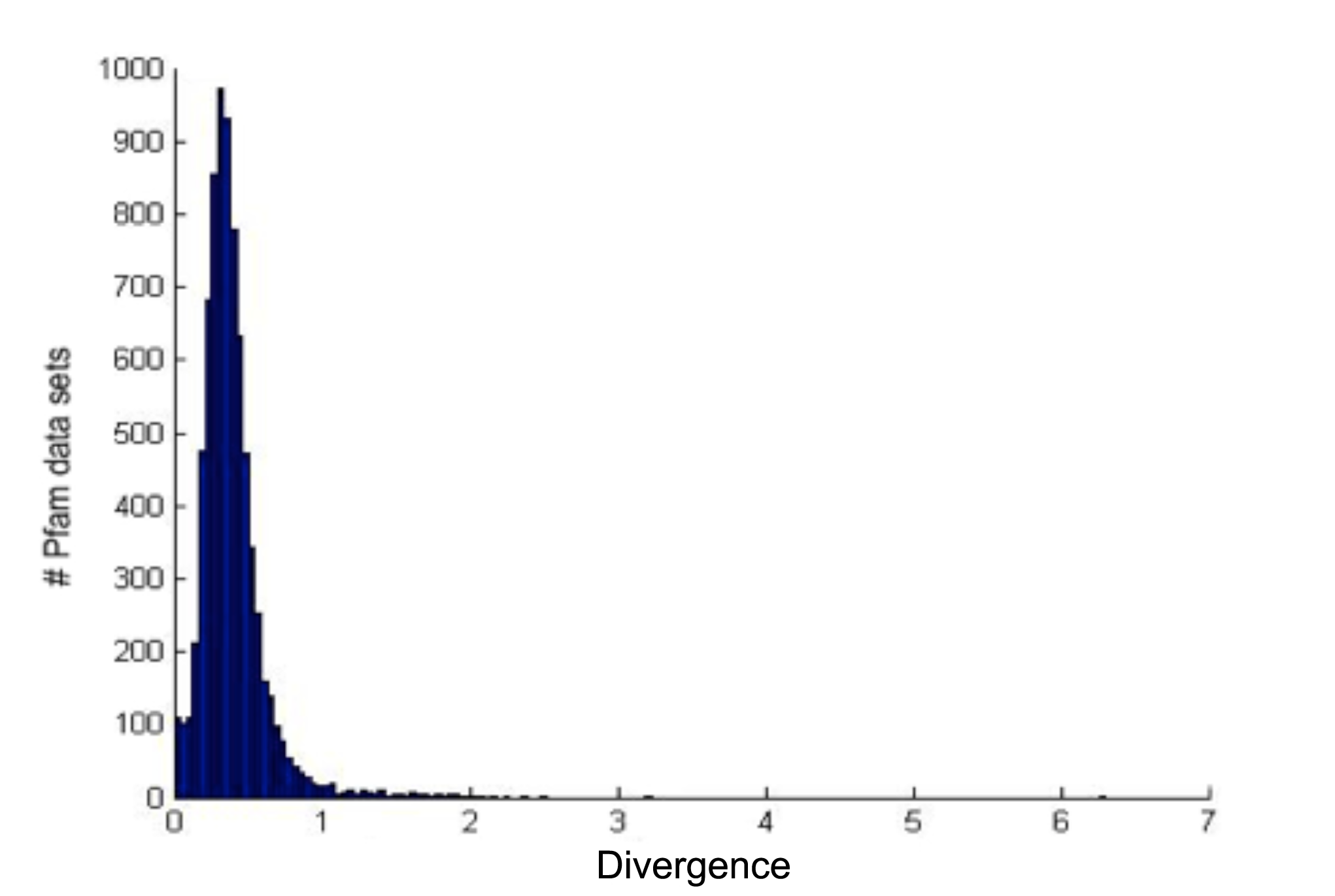

Supplement: Figure S1 — Histogram of PANDIT data sets divergence. The divergence (expected substitutions per amino acid site per branch) was calculated as AA tree length divided by 2*T-3, where T is the number of sequences in the PANDIT data set. The AA tree length and the number of sequences in the each data set were extracted from PANDIT. (TIF) [file pone.0095034.s001.tif]

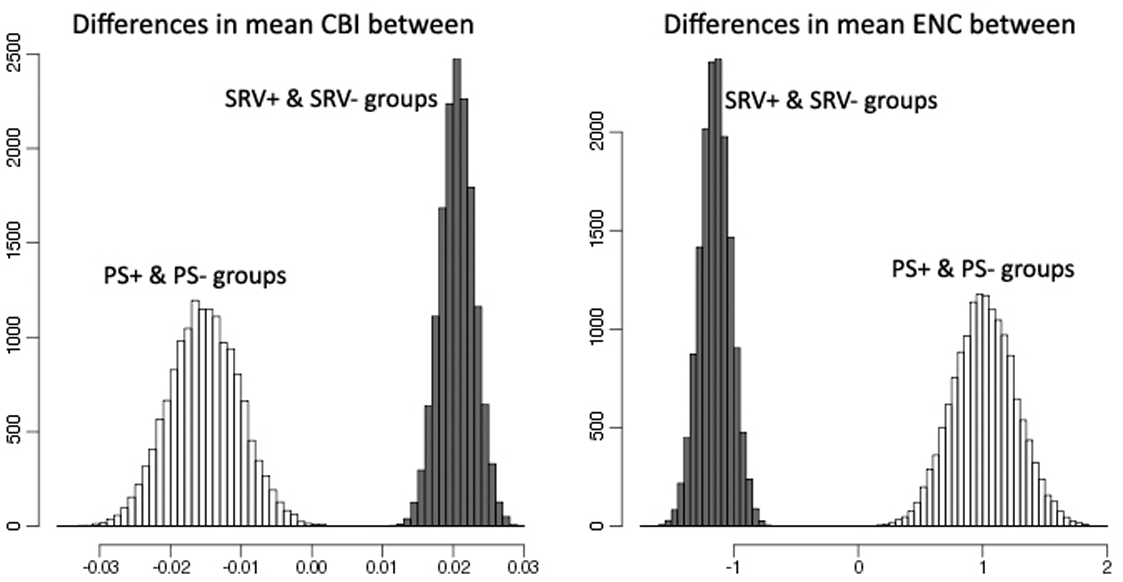

Supplement: Figure S2 — Bootstrap distributions of the differences in mean values of A) Codon Bias Indices (CBI) and B) Effective Number of Codons (ENC) between protein groups showing evidence for site-to-site variation in synonymous rates (SRV+) and those failing to show such evidence (SRV−), and protein groups showing evidence for positive selection (PS+) and those failing to show such evidence (PS−). The differences are significant, since 95% of the histogram area does not include the zero value for all histograms. (TIF) [file pone.0095034.s002.tif]

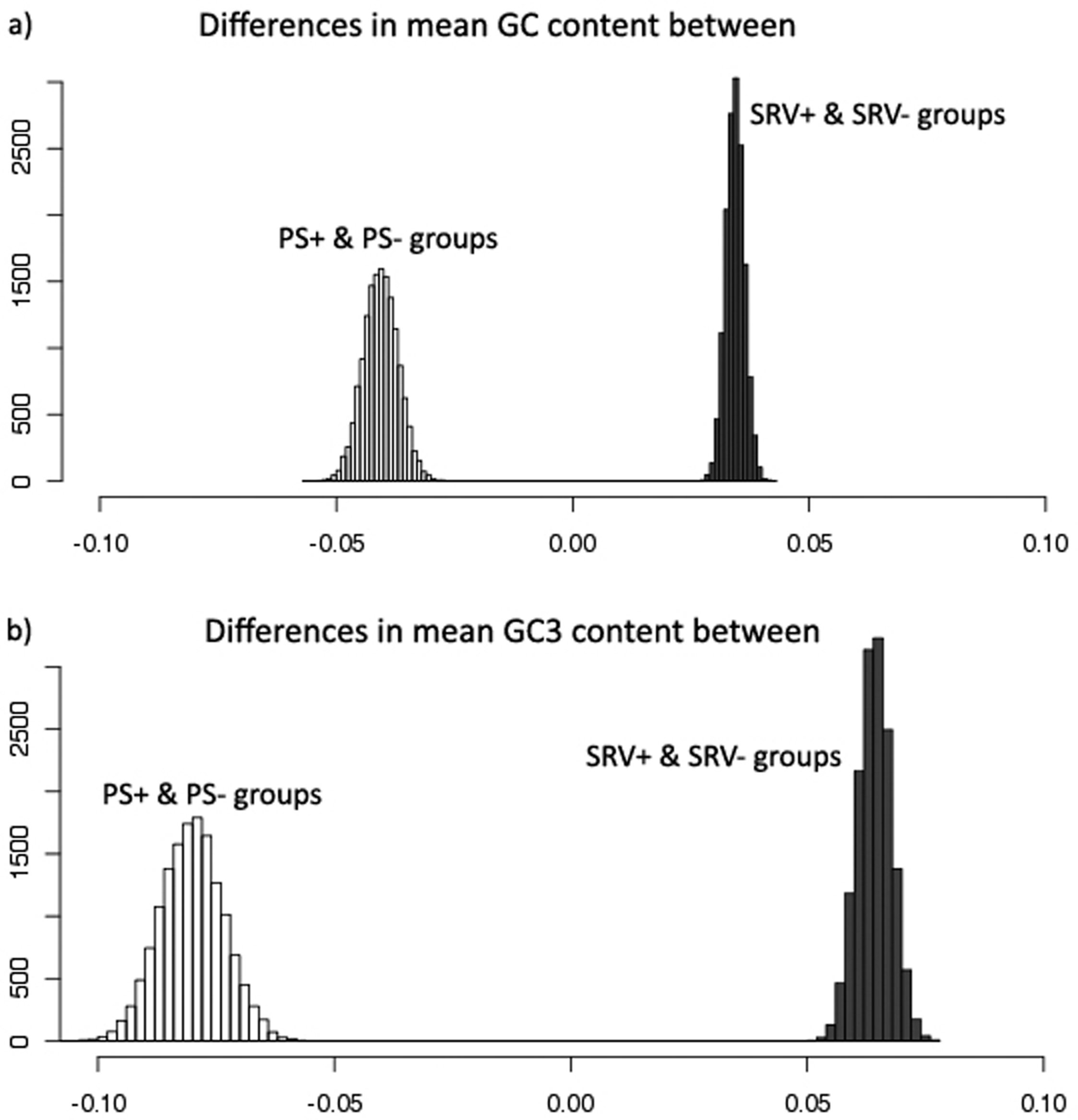

Supplement: Figure S3 — Bootstrap distributions of the differences in A) mean GC content values and B) GC3 content values between PANDIT members showing evidence for site-to-site variation in synonymous rates (SRV+) and those failing to show such evidence (SRV−), and PANDIT members showing evidence for positive selection (PS+) and those failing to show such evidence (PS−). All the differences are significant, since 95% of the histogram area does not include the zero value for all histograms. (TIF) [file pone.0095034.s003.tif]

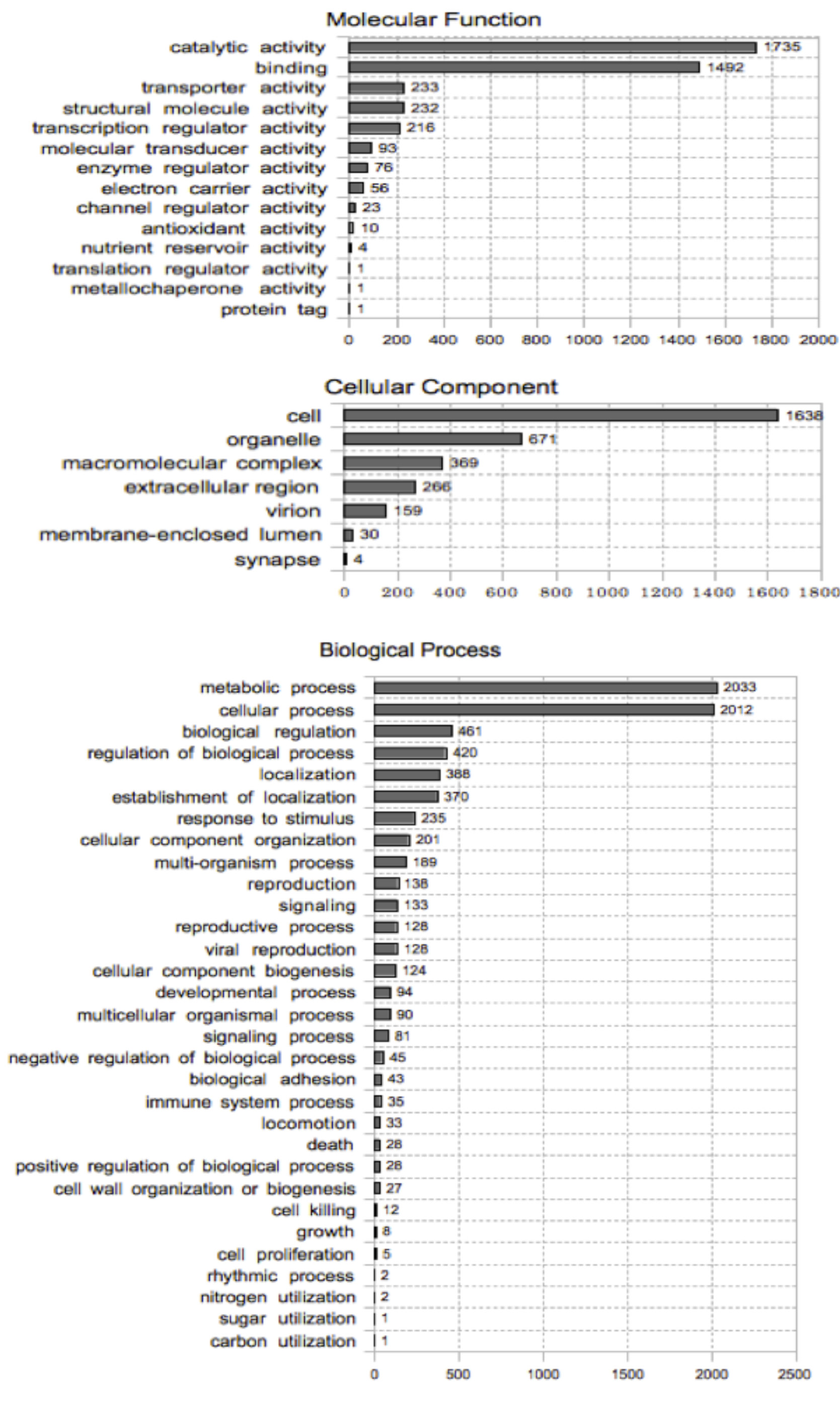

Supplement: Figure S4 — Distributions of data in GO terms. (TIF) [file pone.0095034.s004.tif]

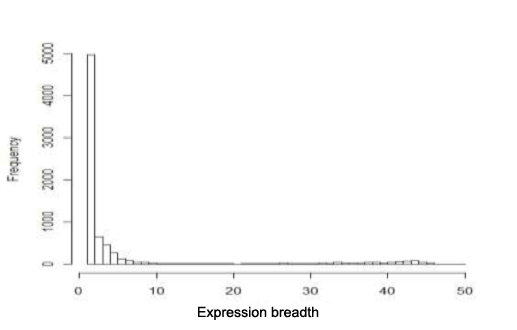

Supplement: Figure S5 — Expression breadth histogram of genes in HumanProteinpedia Database. (TIF) [file pone.0095034.s005.tif]

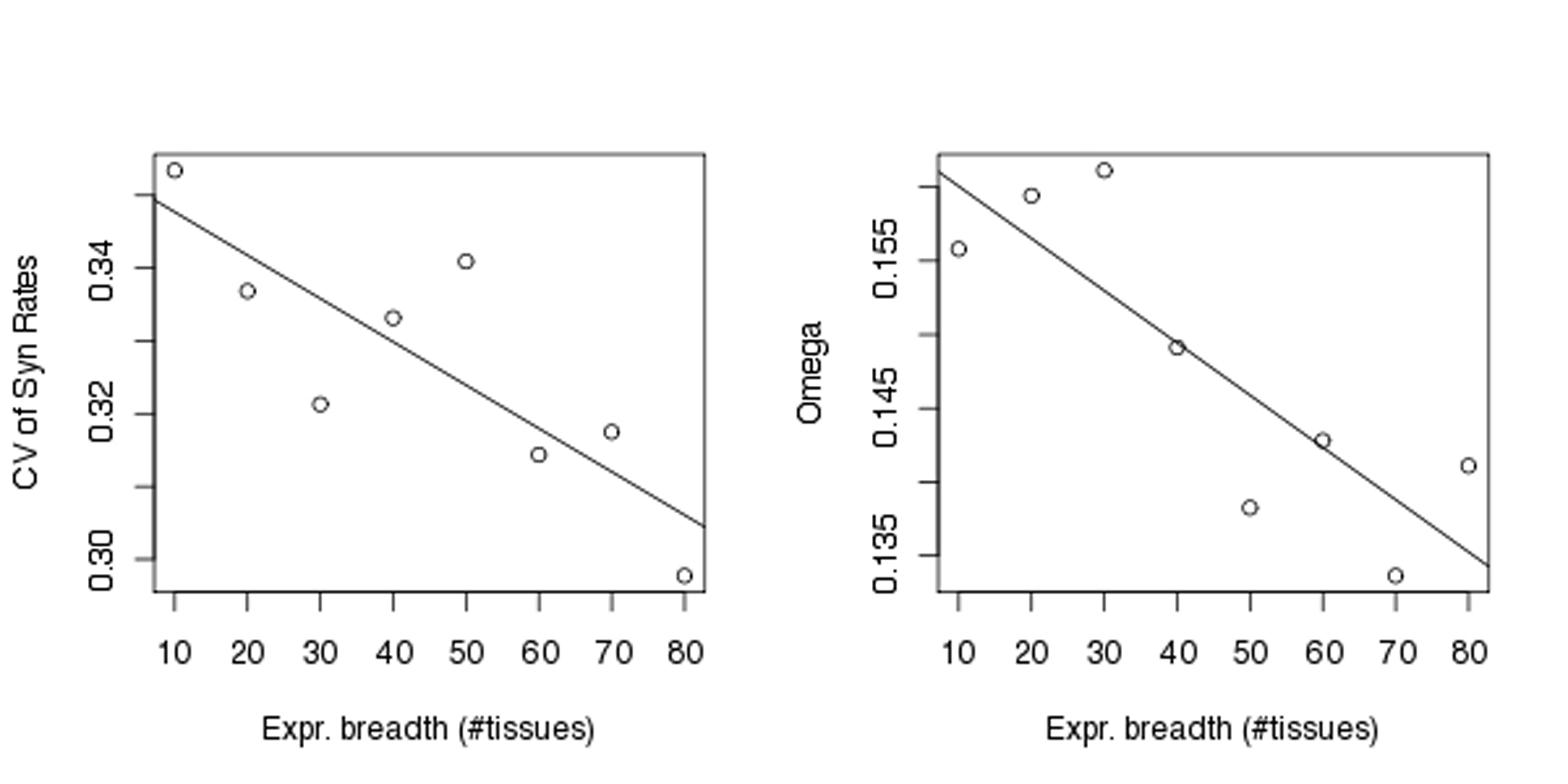

Supplement: Figure S6 — Correlation between gene expression breadth (number of tissues of gene expression) calculated from human Gene Atlas microarray data and A) average CV of synonymous rates and B) average ω ratio, calculated for each bin of 10 tissues. The Gene Atlas microarray expression breadth values were taken from Necsulea et al. (2009). (TIF) [file pone.0095034.s006.tif]

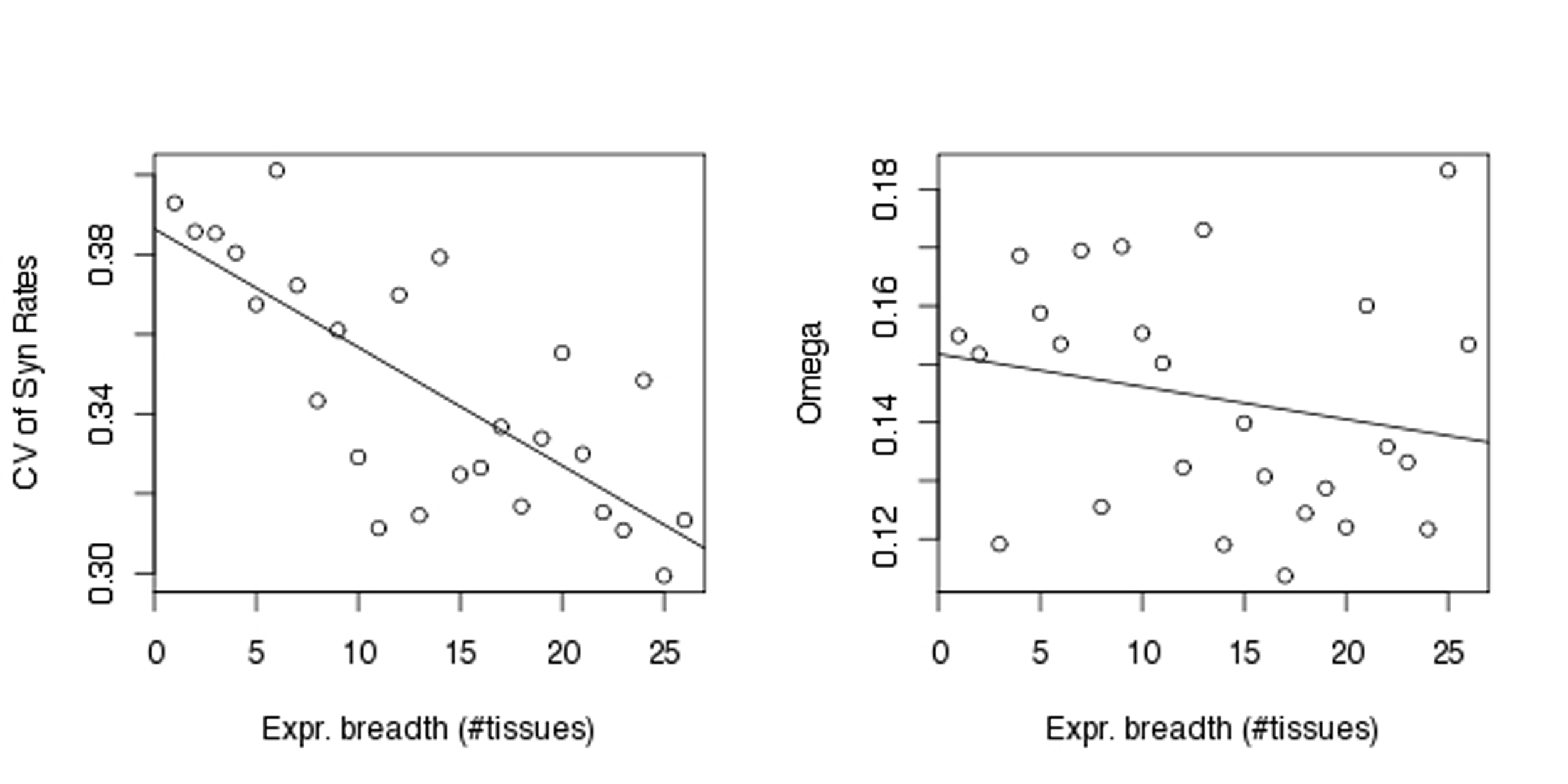

Supplement: Figure S7 — Correlation between gene expression breadth (number of tissues of gene expression) calculated from human SAGE data and A) average CV of synonymous rates and B) average ω ratio. The SAGE gene expression breadth values were taken from Necsulea et al. (2009). (TIF) [file pone.0095034.s007.tif]

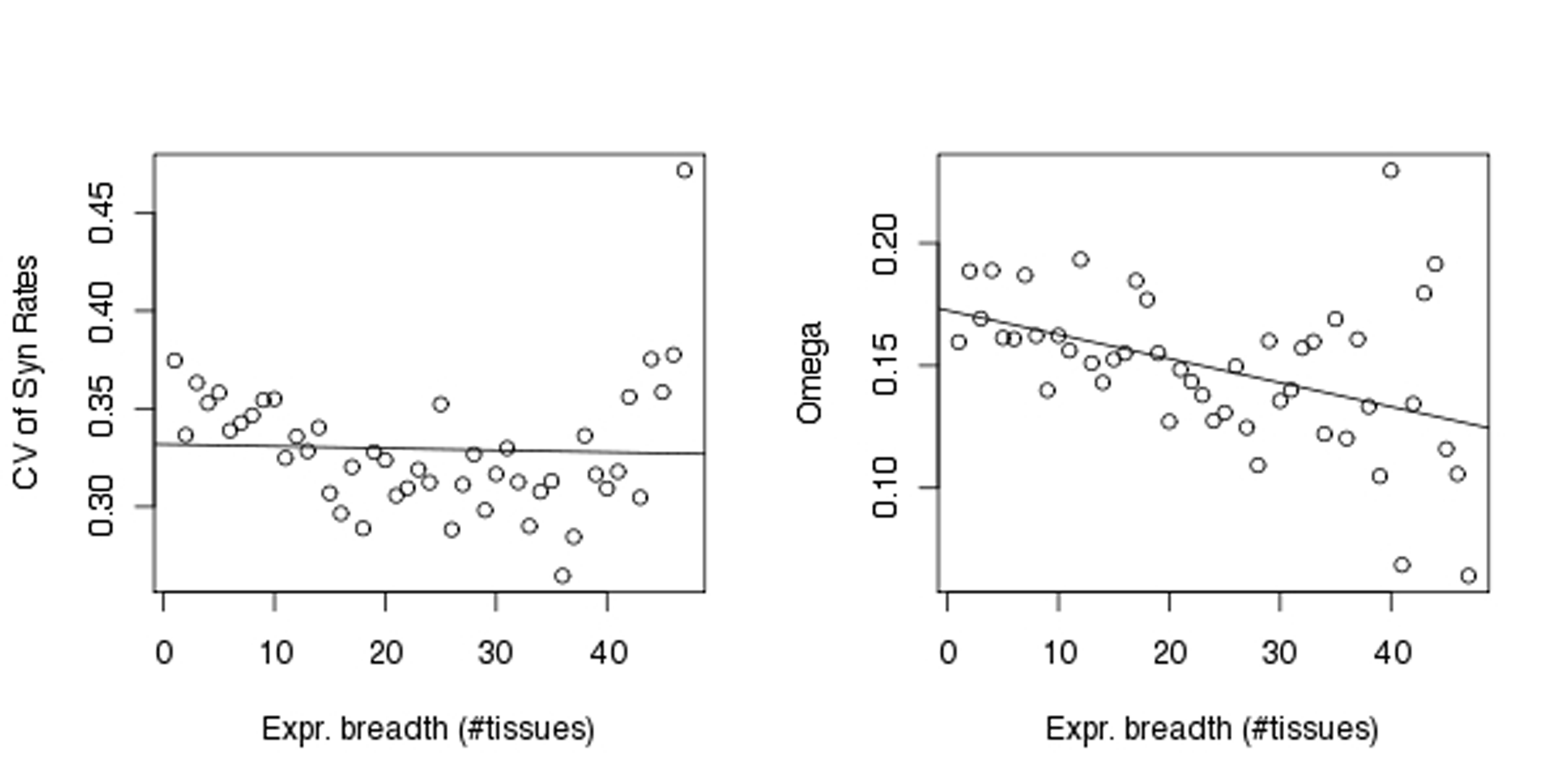

Supplement: Figure S8 — Correlation between gene expression breadth (number of tissues of gene expression) calculated from human EST data and A) average CV of synonymous rates and B) average ω ratio. The EST gene expression breadth values were taken from Necsulea et al. (2009). (TIF) [file pone.0095034.s008.tif]

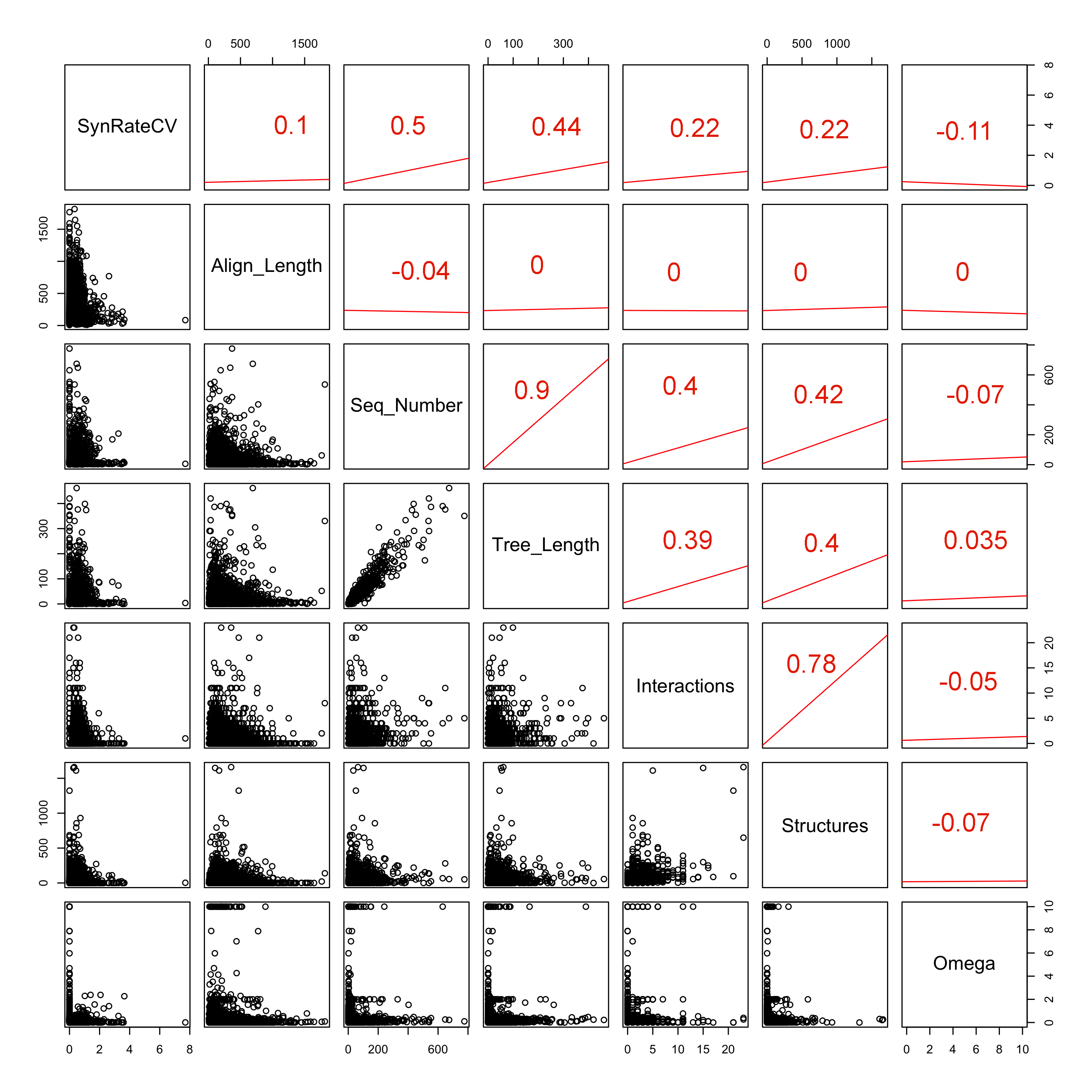

Supplement: Figure S9 — Correlation between individual variables (stated in the diagonal). The numbers in the upper-diagonal plots denote the correlation coefficients for the corresponding pairs of variables. The lower-diagonal plots represent plots of the corresponding data. (TIF) [file pone.0095034.s009.tif]
