# Supplementary material for: Correction: Unraveling Patterns of Site-to-Site Synonymous Rates Variation and Associated Gene Properties of Protein Domains and Families
Source: PLoS One. 2014 Jul 8;9(7):e102721. doi: 10.1371/journal.pone.0102721 (PMC4087007; doi:10.1371/journal.pone.0102721)
Supplement: File S2 — Republished, corrected article. (PDF) [file pone.0102721.s002.pdf]

OPEN ACCESS Freely available online

PLOS ONE

Unraveling Patterns of Site-to-Site Synonymous Rates Variation and Associated Gene Properties of Protein Domains and Families

Slavica Dimitrieva[1,2,3], Maria Anisimova[2,3]*¤

1 Swiss Institute for Experimental Cancer Research (ISREC) and Swiss Federal Institute of Technology Lausanne (EPFL), Lausanne, Switzerland, 2 Department of Computer Science, ETH Zürich, Zurich, Switzerland, 3 Swiss Institute of Bioinformatics (SIB), Lausanne, Switzerland

Abstract

In protein-coding genes, synonymous mutations are often thought not to affect fitness and therefore are not subject to natural selection. Yet increasingly, cases of non-neutral evolution at certain synonymous sites were reported over the last decade. To evaluate the extent and the nature of site-specific selection on synonymous codons, we computed the site-to-site synonymous rate variation (SRV) and identified gene properties that make SRV more likely in a large database of protein-coding gene families and protein domains. To our knowledge, this is the first study that explores the determinants and patterns of the SRV in real data. We show that the SRV is widespread in the evolution of protein-coding sequences, putting in doubt the validity of the synonymous rate as a standard neutral proxy. While protein domains rarely undergo adaptive evolution, the SRV appears to play important role in optimizing the domain function at the level of DNA. In contrast, protein families are more likely to evolve by positive selection, but are less likely to exhibit SRV. Stronger SRV was detected in genes with stronger codon bias and tRNA reusage, those coding for proteins with larger number of interactions or forming larger number of structures, located in intracellular components and those involved in typically conserved complex processes and functions. Genes with extreme SRV show higher expression levels in nearly all tissues. This indicates that codon bias in a gene, which often correlates with gene expression, may often be a site-specific phenomenon regulating the speed of translation along the sequence, consistent with the co-translational folding hypothesis. Strikingly, genes with SRV were strongly overrepresented for metabolic pathways and those associated with several genetic diseases, particularly cancers and diabetes.

Citation: Dimitrieva S, Anisimova M (2014) Unraveling Patterns of Site-to-Site Synonymous Rates Variation and Associated Gene Properties of Protein Domains and Families. PLoS ONE 9(6): e95034. doi:10.1371/journal.pone.0095034

Editor: Tamir Tuller, Tel Aviv University, Israel

Received May 8, 2013; **Accepted** March 23, 2014; **Published** June 4, 2014

Copyright: © 2014 Dimitrieva, Anisimova. This is an open-access article distributed under the terms of the Creative Commons Attribution License, which permits unrestricted use, distribution, and reproduction in any medium, provided the original author and source are credited.

Funding: SD was supported by the Swiss State Secretariat for Education and Research and has received an ETH medal for her Master thesis (including some work presented in this article). MA is supported by the ETH Zurich and is receiving funding from Swiss National Science Foundation (31003A_127325/1). The funders had no role in study design, data collection and analysis, decision to publish, or preparation of the manuscript.

Competing Interests: The authors have declared that no competing interests exist.

* Email: maria.anisimova@zhaw.ch

¤ Current address: Institute of Applied Simulations, Zürich University of Applied Sciences (ZHAW), Wädenswil, Switzerland

Introduction

Synonymous mutations in protein-coding genes preserve an encoded amino acid (AA), and so by Anfinsen's principle [1], should not affect the protein product. Presumably having no fitness effect, synonymous mutations therefore should be invisible to natural selection. However, it has long been suggested that translational selection on synonymous codon usage may act to adapt to organism's tRNA pools [2,3]. In many genes and organisms, differences in abundance of cognate tRNAs for different synonymous codons lead to selection pressure to maximize translation rate in favor of codons that that are read by the most abundant tRNA [4,5,6]. Therefore, the key signature of translational selection is the codon bias in favor of optimal codons affecting whole genes, where fast accurate translation ensures high levels of expression. More recently, experimental studies showed that rare codons may also be favored and selection could act differentially at different synonymous sites, even within the same gene. For example, rare codons may be more frequent in genes with low level of expression, if slow translation is more favorable [7], or involved in regulating expression levels over the time course [8]. Overall, several stages prior to translation involved in protein production may be sensitive to codon choice [9]. Today overwhelming evidences indicate that synonymous mutations can be under site-specific selection on synonymous codon choice. Synonymous mutations can affect splicing control elements, such as exonic splicing enhancers and silencers [10,11] and even can create new 'cryptic' splice sites [12], and so will be affected by selection to avoid codons that could be incorrectly identified as intronic ends. To ensure correct splicing, selection may constrain the synonymous rates of evolution in domains associated with splice control [13,14] and in alternatively spliced exons [15,16,17]. Constraints on synonymous changes help to ensure efficient binding of microRNA to sense mRNA as a mode of gene regulation [9]. Plenty of studies indicate that synonymous mutations can have direct effect on mRNA structure stability, often causing drastic phenotypic effect [18,19,20]. Perhaps even more surprisingly, synonymous mutations can affect the protein folding. Kimchi-Sarfaty and colleagues [21] demonstrated that a synonymous change in the multidrug resistance-1 gene (MDR-1)

causes protein misfolding. The protein with the new altered form helps the cancer cells to get rid of the chemotherapy drug much more efficiently, making the drug useless [21]. Indeed, the folding of a peptide chain is somewhat speed-dependent, and slower production influences the final 3D form of the protein product. Translational pausing due to the usage of rare codons explains why stretches of rare codons were found to correlate to turns, loops and links between protein domains [22,23].

In sum, it is now evident that synonymous mutations can be under a variety of selective mechanisms. With over 40 genetic diseases (including cancers and diabetes) associated with synonymous mutations, it is now clear that such mutations can have important fitness consequences, unlike previously thought [24,25]. Chamary and Hurst [26] estimated that 5–10% of human genes contain at least one region where silent mutations could be harmful. Based on the analysis of human genetic associations of SNPs with disease, Chen et al. [27] concluded that non-synonymous and synonymous SNPs show similar likelihood and effect size of human disease association. Finally, synonymous mutations may be responsible for individual differences in disease susceptibility and treatment outcomes (see [25] for a comprehensive review).

Recently, many large-scale statistical studies focused on detecting pervasive positive diversifying selection on the protein, as measured by the nonsynonymous to synonymous rates ratio $\omega = d_N/d_S$ [28]. However, patterns of selection on synonymous codons are poorly understood. Most often negative selection on synonymous codons is studied by measuring the average codon usage per gene. Resch et al. [29] performed a large-scale scan for positive selection on synonymous sites, where average pairwise synonymous substitution rate d_S for a gene was compared to the corresponding average intron rate in mouse-rat gene pairs. This approach found that positive selection on synonymous sites could be even more frequent than positive selection on the protein. However, the pairwise averaging approach typically lacks power [30] and overlooks the impact of site-specific synonymous rate variation (SRV) over the protein-coding sequence. Zhou et al. [31] proposed to distinguish synonymous rates of change between different types of synonymous codons ("preferred" and "un-preferred"). Applied to yeast and worm genes, their method found substantially lower number of genes with positive selection on synonymous sites compared to [29]. Clearly, the accuracy of such an approach would be affected by uncertainties in identifying preferred and un-preferred codons. But perhaps more importantly, the method of Zhou et al. [31] models only average synonymous rates per gene and so cannot capture site-specific selection pressure that acts on the DNA or mRNA level related to transcription, splicing, expression regulation or mRNA structure stability. Significant variation of synonymous rates (d_S) reflects that the evolutionary forces act differently at different synonymous sites, likely due to variation in selective constraints. Thus candidate genes affected by either purifying or positive selection on the DNA can be detected with a systematic analysis of the SRV, using the extent of d_S variation as a proxy for selection.

Here for the first time we present a large-scale analysis of homologous proteins – with the aim to improve our understanding of the nature of synonymous changes and the SRV in protein-coding sequences. In contrast to the study of Resch et al. [29], we analyzed multiple sequence alignments (where evolutionary information is at the maximum) using Markov codon models with SRV. We determined how often and where strong SRV occurs, and listed the gene properties that make the SRV more likely. The patterns of SRV and groups of genes enriched with SRV may provide important clues for other studies focusing on understanding disease, optimizing transgene design, as well as those dedicated to determining specific and general evolutionary trends in molecular sequences. Our study opens directions for exploring new measures of selective pressure that incorporate the effect of selection on synonymous sites.

Materials and Methods

The Data

7738 homologous groups and corresponding alignments of protein-coding DNA and AA sequences were obtained from the PANDIT database v17.0 [32]; http://www.ebi.ac.uk/goldman-srv/pandit). PANDIT contains protein domains and families, derived from the Pfam-A seed alignments [33]. Phylogenetic trees were inferred for each homologous group by maximum likelihood (ML) under the amino acid model LG+Γ+F, as implemented in PhyML3.0 [34]. These ML estimates of trees were consequently used for all optimizations under codon models (see below). To avoid drawing conclusions based on saturated alignments, we removed groups where the average divergence was greater than two expected substitutions per amino acid site per branch (Figure S1). Annotations for each homologous group were taken from the PANDITplus database [35]; http://panditplus.org), an extension of PANDIT, integrating data from a variety of reliable and curated bioinformatics sources. It provides access to data on protein interactions, functional and chemical pathway annotation, gene expression and association with diseases. The estimates from evolutionary codon models computed for this study are now also available from PANDITplus.

Analyses of Positive Selection (PS) on the Protein and the Synonymous Rate Variation (SRV)

Pervasive diversifying positive selection (PS) on the protein was evaluated by ML using Markov models of codon evolution, as implemented in the codeml program from the PAML package v4.1 [36]. The selective pressure at the protein level was measured by the ω-ratio, with $\omega<1$, $=1$, or >1 indicating purifying, neutral or positive selection on the protein respectively [37]. For each homologous group we computed estimates of the average ω using model M0, which assumes constant selective pressure across codon sites and over time. ML estimates of branch lengths under M0 were then used as starting (or fixed) values in all following computations under codon models. Likelihood ratio test (LRTs) of nested codon models M0 vs M3, and M7 vs M8 was used to determine whether a gene was affected by selection [38,39,40]. Evidence for adaptive evolution in a gene was considered sufficient if the following conditions were met: (1) both LRTs were significant at 5% level with an estimated $\omega>1$, (2) the estimated proportion of positively selected sites was large enough to include at least one site, and (3) the SRV-aware model (DUAL, [41]) supported the presence of PS. Condition (3) was required to avoid a potential bias on the detection of PS as a result of SRV. Groups of proteins with evidence of PS are further refereed to as PS+, while those with no such evidence are denoted as PS−.

To determine whether a gene exhibited site-to-site SRV, we applied an LRT between a codon model where d_S was assumed constant (model M3) and a model where both d_S and d_N could vary (DUAL model) [41]. ML optimization for this task was performed with the HYPHY program [41]. Both d_S and d_N were assumed to be drawn from independent general discrete distributions, each with three rate categories. Evidence for site-to-site SRV was considered sufficient if: (1) the LRT was significant at 5% level and (2) the coefficient of variation (CV) of the synonymous rates was $>$ 0. The second condition was added to exclude the few cases with

artifacts of ML estimation, where the LRT showed significantly better fit of the model with variable d_S, but yet the estimated CV of d_S was 0. Data classified as having significant SRV is further referred to as SRV+ set, while data where d_S can be assumed constant is further referred to as SRV−.

Patterns in 7341 data sets were analyzed, after filtering out protein groups that were too diverged or had convergence problems during ML optimizations. To avoid optimization problems each analysis was performed multiple times and one with a higher log-likelihood was selected.

Analyses of Over/Under-representation in Functional Categories

GO and KEGG annotations for each group were obtained from PANDITplus [35]. To account for the hierarchical nature of GO and KEGG data, each gene (protein) was considered to belong to all parent categories where it was directly assigned. To test the over/under-representation of genes with specific feature (PS or SRV), the data sets were divided into two groups: those showing evidence for the feature of interest (PS+, SRV+) and those that failed to show such evidence (PS−, SRV−). For each tested functional category *C*, a 2×2 contingency table was constructed containing the numbers of genes assigned and not assigned to *C*. To test for independence of rows and columns one-sided *P*-values were computed using Fisher's exact test. As test sets overlapped, the raw *P*-values from Fisher's exact test were adjusted to control the false discovery rates [42].

Codon Bias, Autocorrelation and Nucleotide Composition

For each protein group, we computed total GC content, GC content at third codon positions (GC3), and codon usage indices CBI (Codon Bias Index, [43]) and ENC (Effective Number of Codons, [44]), using the CodonW program [45]. CBI measures the usage of optimal codons, ranging between 1 (only optimal codons are used) to −1 (only non-optimal codons are used), with 0 for random codon choice. ENC is another measure of synonymous codon usage, ranging between 20 (only one codon is used for each AA) and 61 (codons are used randomly).

Finally, we computed the TPI (tRNA Pairing Index), a statistical measure of tRNA reusage [46,47], using the dedicated Darwin functions [48]. By definition, the TPI ranges from −1 for perfectly anticorrelated tRNA changes (i.e maximal number of tRNA changes) to +1 for perfectly autocorrelated (minimal number of tRNA changes). For example, in a sequence where one AA is encoded by two tRNAs X and Y, highly autocorrelated case is XXXXYYYY, while XYXYXY is highly anticorrelated case. For a comprehensive review of codon usage measures see [49].

Note that when measuring the correlation between any two phenomena, we computed both Spearman and Pearson correlation coefficients, which provided very similar results. We therefore show only the Spearman correlation values.

Analyses of Gene Expression Data

Several sources of gene expression data were used in this study. Mappings of gene expression in human tissues (data from HumanProteinpedia [50]) were obtained from PANDITplus. These data do not contain information on the expression levels, but only inform whether a gene is expressed in a certain human tissue or not. Fisher's exact tests were performed to identify the tissues with over/under-representation of expressed genes with SRV and PS. Information on human gene expression breadth of Ensembl genes from three types of experiments (Gene Atlas microarray, EST and SAGE) was taken from [51]. These data provide information on the gene expression breadth measured by the number of tissues where the gene is expressed, but no information about the expression levels or the tissue of expression. Ensembl gene IDs were mapped to Pfam IDs using BioMart module of the Ensembl database v.62 [52]. Note that in the expression data analyses we used gene-Pfam mappings derived from gene associations with full Pfam alignments. We also analyzed expression data from Gene Atlas U133A Affymetrix microarray from the BioGPS portal of the Genomics Institute of the Novartis Research Foundation ([53]; http://biogps.gnf.org/downloads), mapping individual protein sequences from the seed PANDIT alignments to microarray probes. We used these data to analyze gene expression levels by calculating the distribution of the log expression values for the categories of interests.

Clustering Analyses

Hierarchical clustering of gene categories was performed for KEGG pathways. The dissimilarity matrix for the clustering was defined so that any two categories A and B from the same hierarchical level had dissimilarity $d_{AB} = 0$ when all SRV+ genes were assigned to both categories A and B, and dissimilarity $d_{AB} = 1$ when A and B did not share any SRV+ gene. More specifically, dissimilarity between two categories A and B was defined as:

$$d_{AB} = \frac{1 - |N(A) \cap N(B)|}{minN(A),N(B)}$$

where N(X) denotes the number of SRV+ genes in category X.

Results

Significant SRV was found in 42% (or 37%) of protein groups at 5% (or 1%) significance level. This suggests that the phenomenon of site-to-site heterogeneity of synonymous rates is widespread and deserves attention. Extreme SRV was detected in 154 datasets (CV≥1, see Table S1). Notably, certain Pfam clans were exclusively composed of SRV+ groups. Recall that clans are higher-level clusters of related families, grouped based on structure, function, matching of families HMMs and profile-profile comparisons. The list of SRV exclusive clans includes p53-related proteins and ABC transporters (see Table S2).

Note that PS on the protein was detected in 11% (or 7%) of groups at 5% (or 1%) significance level (consistent with previous estimates, eg. [54]). We observed weak but significant negative correlation ($\rho = -0.11$, $P < 10^{-16}$) between the variability of synonymous rates and the average ω-ratio across protein sites. This indicates that proteins that are more conserved tend to have greater SRV among sites. A bootstrap analysis on the differences in mean ω for protein groups classified as SRV+ and SRV−, confirmed that proteins with SRV tend to be under stronger purifying selection (lower ω) compared to proteins where synonymous rates may be assumed constant (Figure 1A).

Reflecting Pfam, our protein groups included protein families (74%), domains (23%), motifs (1%) and repeats (2%). SRV was significantly overrepresented in protein domains, but underrepresented in protein families (Table 1). An opposite pattern was observed for PS: protein domains showed significant underrepresentation of groups with PS, while protein families were overrepresented with PS+ groups. Motifs and repeats did not show any significance for over or underrepresentation with SRV+ or PS+ groups, most likely due to their small dataset numbers and short sequences, which increased variance of ML estimates.

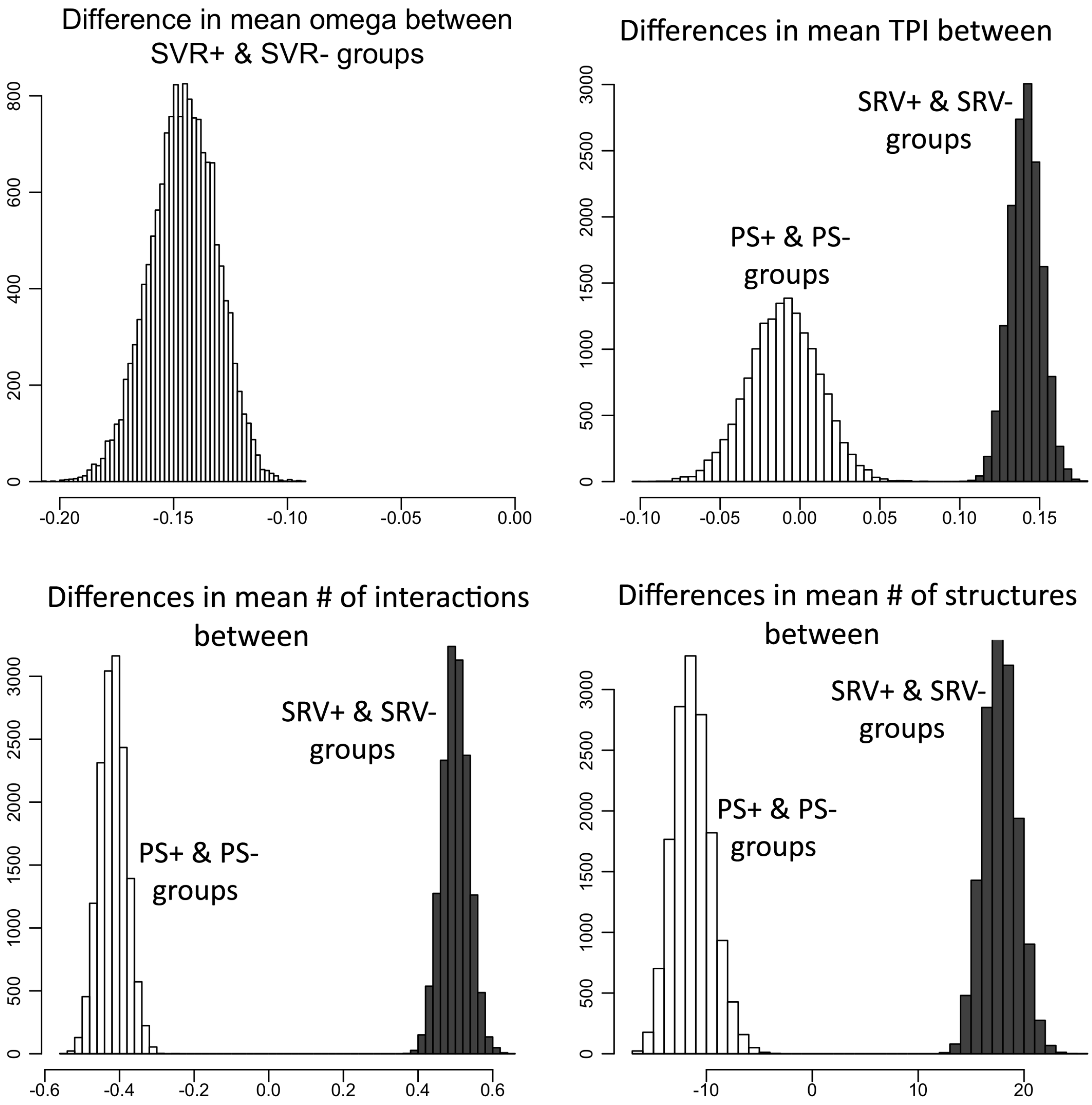

Figure 1. Bootstrap distribution of the differences in A) the mean ω-ratio, B) tRNA reusage, measured through tRNA Pairing Index (TPI), C) number of interactions and D) number of structures, between protein groups having site-to-site variation in synonymous rates (SRV+) and protein groups having constant synonymous rates (SRV−). The plots B), C) and D) also show the bootstrap distributions of the corresponding differences between protein groups showing evidence for positive selection (PS+) and those failing to show such evidence (PS−). All differences (except for TPI in PS+/PS− data) are significant since 95% of the histogram area does not include the zero value.
doi:10.1371/journal.pone.0095034.g001

Table 1. Overrepresentation (+) and underrepresentation (−) of SRV and PS in different data categories.

Pfam type	SRV		PS	
	**Representation**	**P-value**	**Representation**	**P-value**
Protein Domains	+	10^{-33}	−	10^{-9}
Protein Families	−	10^{-28}	+	10^{-10}

doi:10.1371/journal.pone.0095034.t001

Dependencies between Site-to-site SRV and Gene Properties

Selection for translational speed favors codons matching the cognate tRNA profile. We investigated whether the codon bias and tRNA reusage could contribute to the observed site-to-site SRV. CV of synonymous rates was correlated with both codon bias and tRNA reusage (Table S3). In the SRV+ group the average codon bias and tRNA reusage were significantly larger than in the SRV− group (Figure 1B; Figure S2, Table 2). In contrast PS+ group had on average weaker codon bias compared to the PS− group (Table 2, Figure S2).

It has been suggested that selection at synonymous sites favors high GC, which is reflected in a correlation between codon bias and GC3, the GC content at third codon positions [55]. Some studies reported that GC at synonymous sites was higher than in the flanking introns [56,57], and that GC content could contribute to the regulation of splicing signals, in which case synonymous mutations may lead to exon skipping associated with disease [58]. These evidences indicate the possibility of selection acting on synonymous sites. In our data we observed that the variability of d_S correlated positively with the variation of GC and GC3 among homologous genes, but not very well with the GC and GC3 content (see also Figure S3 and Table S3).

Our results suggest that proteins with many interactions evolved under stricter purifying selection (Figure 1C), which is in agreement with the extended complexity hypothesis [54]. We observed positive correlation between the number of interactions and CV of d_S ($\rho = 0.22$, $P < 10^{-16}$; Figure 1C). Since SRV and PS groups were unequally represented within different data types (domains, families, motifs and repeats), bootstrap analyses were repeated for each data type separately. The reported trends were significant for domains, families and repeats. Further, proteins forming many structural complexes exhibited stronger SRV (Figure 1D) and tended to be more conserved and less likely to be under recurrent diversifying positive selection. We observed positive correlation between the number of structural complexes that proteins can form and the CV of d_S ($\rho = 0.22$, $P < 10^{-16}$), and weak negative correlation with the ω-ratio ($\rho_{\text{Spearman}} = -0.08$, $P < 10^{-12}$; Pearson correlation was not significant).

Overall, our data show that there is a correlation between the individual variables, most notable between GC and GC3 content ($\rho = 0.92$); codon bias and GC3 content ($\rho = 0.74$); codon bias and GC content ($\rho = 0.73$); number of interactions and number of structures ($\rho = 0.52$); codon bias and codon autocorrelation ($\rho = 0.41$); GC content and codon autocorrelation ($\rho = 0.23$) etc. However, some of these variables could be independently associated with one another. For instance, it has been widely reported that codon bias is associated with various biological factors, such as gene expression level, tRNA abundance, GC composition, protein structure etc. Furthermore, it was shown that the similarity in codon usage is a strong predictor of protein-protein interactions [59]. To get more insights, we conducted a multivariate analysis and sought to find the individual variables that give the greatest separations between the SRV+ and SRV− groups. We quantified the "separation" F between the SRV+ and SRV− groups achieved by a particular variable (ω-ratio, CBI, TPI, GC/GC3 content, #interactions, #structures) as the ratio of its "between-groups" variance to its "within-groups" variance. Surprisingly, the greatest separation between the two groups was achieved based on the number of protein-protein interactions (F = 218), followed by the tRNA reusage index (F = 193), the number of protein structures (F = 146), the ω-ratio (F = 74), codon bias (F = 68), GC3 content (F = 30) and GC content (F = 16). Finally, we performed principal component analysis (PCA) to investigate whether most of the variation between our SRV+/SRV− data can be captured using principal components that were linear combinations of all or some of the other variables (ω-ratio, CBI, TPI, GC/GC3 content, #interactions, #structures). The first two principal components (PC) explain 70% of the variance of SRV. The first PC (explaining 45% of the variance) represented a contrast between the ω-ratio and the other variables (CBI, TPI, GC content, GC3 content, #interactions, #structures), with the largest loadings (in absolute) values for GC3 content (0.59), GC content (0.59) and CBI (0.55), the loadings of the other components were <0.06. This supports the negative correlation between CV of SRV and the ω-ratio, and its positive correlation with all the other variables, but suggests that omega has little impact (based on the low loading value). The second PC represents a contrast between the ω-ratio, CBI, TPI, #interactions and #structures, and the variables GC content and GC3 content. The largest loadings of this PC were for #interactions (0.7), #structures (0.7), while the loadings (in absolute) values of the other variables were <0.08. Overall, the PCA demonstrates that the influence of the above-mentioned factors on SRV is complex due to the strong dependencies among them.

Table 2. Differences between the mean values of the attribute (#interactions, #structures, codon bias and tRNA reusage) in SRV+ and SRV− data, and in PS+ and PS− data correspondingly.

Attribute	Difference between attribute means in SRV+ and SRV− data (median [IQR])	Difference between attribute means in PS+ and PS− data (median [IQR])
**Interactions**	0.50 [0.48, 0.53	−0.42 [−0.44, −0.39]
**Structures**	17.72 [16.65, 18.80]	−11.38 [−10.12, −12.59]
**Codon bias (CBI measure)**	0.02 [0.019; 0.022]	−0.01 [−0.018; −0.011]
**Codon bias (ENC measure)**	−1.3 [−1.39; −1.22]	1.0 [0.84; 1.18]
**tRNA reusage**	0.14 [0.13; 0.15]	−0.01 [−0.02; 0.005]
**GC content**	0.042 [0.04; 0.043]	−0.041 [−0.038; −0.043]
**GC3 content**	0.08 [0.078; 0.083],	−0.08 [−0.085; −0.076]

All p-values are $<10^{-16}$, except for the differences in mean values of tRNA reusage (TPI) between PS+/PS− data where there was no significance. This table corresponds to Figure 1 and Figure S2.
doi:10.1371/journal.pone.0095034.t002

Site-to-site SRV and Protein Function, Interactions and Reaction Networks

We examined the distribution of GO functional categories [60] with respect to site-to-site SRV. Since our protein groups were unevenly distributed among GO categories (Figure S4), significant over/under-representation was more difficult to detect for sparsely sampled categories, with better power for GO terms annotating larger number of protein groups.

Table 3 summarizes the results of GO-enrichment tests for "Cellular Component". Categories enriched with SRV+ proteins included cell envelope, membrane, wall and external encapsulating structure. Underrepresentation of SRV+ proteins was found in extracellular region, membrane-enclosed lumen and organelles. Our results for PS+ proteins are consistent with previous findings [54,61]: extracellular region and MHC protein complex were found as overrepresented with PS+ proteins, while the cellular components that are mostly internal to the cell, organelles and macromolecular complex were identified as strongly conserved.

Analyses of "Molecular function" categories are summarized in Table 4. Categories enriched with SRV+ proteins included catalytic and transporter proteins, proteins with a role in carrying electrons, or those important for binding (with exception of receptor binding). Underrepresentation of SRV+ proteins was observed among the proteins that participate in receptor binding and enzyme regulation. Categories underrepresented with PS+ proteins included catalytic and transporter proteins, and those with a role in binding.

Enrichment analyses of "Biological process" categories are summarized in Table 5. We found an overrepresentation of SRV+ among the proteins with function in metabolism, cellular processes and in localization and transport. Proteins that participate in multi-organism processes (symbiosis, interaction with host), defensive response to stimulus and reproduction were found as least likely to have significant site-to-site SRV. For PS+ proteins we observed the opposite: proteins involved in metabolic and cellular processes, as well as biological regulation were found to be most conserved and least likely to undergo adaptive evolution. Proteins related to immune system processes and response to stimulus, which represent obvious targets for adaptive evolution, were enriched with PS.

Table 3. Over/under-representation of selective forces in GO categories for Cellular Component.

GO Categories	*SRV*		*PS*		#pfam
	**Over(+)/Under(−) represent.**	**Signif.**	**Over(+)/Under(−) represent.**	**Signif.**	
**cellular component**					
extracellular region	−	***	+	***	**205**
**cell**	+	**	−	**	**1491**
**cell part**	+	**	−	**	**1491**
intracellular			−	**	872
**membrane**	+	*			**717**
**cell wall**	+	*			**29**
**cell envelope**	+	**			**38**
endomembrane system	−	**			**55**
**external encapsulating structure**	+	**			**63**
intracellular part			−	**	773
**extrachromosomal DNA**	+	**			**6**
**ribonucleoprotein complex**	+	*			**116**
virion			+	***	151
virion part			+	**	141
viral capsid			+	*	98
viral envelope			+	*	35
membrane-enclosed lumen	−	**			**25**
organelle lumen	−	**			**23**
intracellular organelle lumen	−	**			**23**
macromolecular complex			−	*	346
ribosome			−	**	98
MHC protein complex			+	**	4
organelle	−	***	−	**	**597**
membrane-bounded organelle	−	***	−	*	**423**
intracellular membrane-bounded organelle	−	***	−	*	**420**
intracellular organelle	−	***	−	**	**593**

Notation: Significance levels are at the 5% (*), 1% (**), or 0.1% (***). Boldface indicates overrepresentation of SRV; italics indicates underrepresentation of SRV.
doi:10.1371/journal.pone.0095034.t003

Table 4. Over/under-representation of selective forces in GO categories for Molecular Function.

GO Categories	SRV		PS		#pfam
	Over(+)/Under(−) represent.	Signif.	Over(+)/ Under(−) represent.	Signif.	
**molecular function**					
**electron carrier activity**	+	*			**53**
**catalytic activity**	+	***	−	***	**1536**
**oxidoreductase activity**	+	**	−	***	**251**
transferase activity	+	*	−	*	444
transferase activity, transferring one-carbon groups			−	**	76
**hydrolase activity**	+	***			**533**
**isomerase activity**	+	***			**58**
**ligase activity**	+	***			**75**
**ligase activity, forming carbon-oxygen bonds**	+	***			**25**
structural molecule activity					220
structural constituent of ribosome			−	**	98
**transporter activity**	+	**	−	*	**221**
**binding**	+	***	−	***	**1286**
**nucleotide binding**	+	***	−	**	**263**
**purine nucleotide binding**	+	***	−	*	**231**
**ribonucleotide binding**	+	***	−	*	**217**
protein binding			−	*	**244**
receptor binding	−	*			**83**
**nucleoside binding**	+	***	−	*	**200**
**purine nucleoside binding**	+	***	−	*	**199**
**nucleic acid binding**	+	**	−	***	**515**
DNA binding			−	**	368
**carbohydrate binding**	+	*			**27**
ion binding			−	**	270
cation binding			−	**	269
**cofactor binding**	+	***			**61**
**coenzyme binding**	+	***			**47**
enzyme regulator activity	−	**			**68**

Notation: Significance levels are at the 5% (*), 1% (**), or 0.1% (***). Boldface indicates overrepresentation of SRV; italics indicates underrepresentation of SRV.
doi:10.1371/journal.pone.0095034.t004

These findings suggest that forces driving either SRV or PS are not independent from the gene function, with distinct biases in their distribution among GO categories. Furthermore, with exception of organelles, there was a visible tendency to observe enrichment with SRV+ proteins in the same GO categories that were underrepresented with PS (Tables 3–5).

Information on biological pathways, in which a protein is involved, includes chemical reactions within a cell whose dependencies and dynamics are distinct from the notion of a biological process as classified by GO. Therefore, we also performed enrichment analyses for 18,041 human genes in KEGG with respect to their biological pathways (Table 6). We classified a KEGG gene as being affected by PS (or SRV), if it was mapped to at least one PANDIT group that was classified as PS+ (or SRV+ respectively).

SRV+ genes were found to be enriched for a wide variety of functions related to metabolic pathways, particularly in carbohydrate and amino acid metabolism, metabolism of cofactors and vitamins, metabolism of xenobiotics by cytochrome and drug metabolism - cytochrome. This finding is consistent with our observations about metabolic processes based on GO. However, the analyses of KEGG pathways also revealed certain metabolic pathways where SRV+ genes were underrepresented. This result might be due to the fact that gene ontologies are not equivalent to pathways: pathways could involve genes that are not directly relevant to the metabolic process, but are included because of the pathway inter-process dependencies and specific dynamics. Additionally, this may be also due to the fact that KEGG analysis is done only on human genes, unlike GO.

Our analyses of GO terms identified that metabolic processes were generally conserved. Studies of positive selection on the protein level [54,61,62] mainly refer to metabolic processes, but not to metabolic pathways. The differences in our results from KEGG and GO for positive selection might be due to the way of classification of KEGG genes as PS+ (having found at least one PANDIT data product of that gene as positively selected). Namely,

Table 5. Over/under-representation of selective forces in GO categories for Biological Processes.

GO Categories	SRV		PS		#pfam
	Over(+)/ Under(−) represen.	Signif.	Over(+)/ Under(−) represent.	Signif.	
**biological process**					
reproduction	−	**	+	*	**130**
**metabolic process**	+	***	−	***	**1807**
oxidation reduction			−	**	98
**nitrogen compound metabolic process**	+	***	−	***	**883**
**amine metabolic process**	+	***	−	*	**127**
**cellular nitrogen compound metabolic process**	+	*	−	***	**840**
**biosynthetic process**	+	***	−	***	**879**
**macromolecule biosynthetic process**	+	*	−	***	**591**
regulation of biosynthetic process			−	**	231
**cellular biosynthetic process**	+	**	−	***	**838**
regulation of metabolic process			−	**	260
**macromolecule metabolic process**	+	***	−	***	**1022**
**gene expression**	+	***	−	***	**62**
**macromolecule biosynthetic process**	+	***	−	***	**591**
**protein metabolic process**	+	***	−	***	**378**
**cellular macromolecule metabolic process**	+	***	−	***	**875**
**cellular metabolic process**	+	***	−	***	**1383**
**organic acid metabolic process**	+	***			**139**
**cellular amino acid and derivative metabolic process**	+	***	−	*	**113**
**cellular nitrogen compound metabolic process**	+	**	−	***	**840**
**cellular ketone metabolic process**	+	***	−	**	**139**
**cellular biosynthetic process**	+	***	−	***	**838**
**cellular macromolecule metabolic process**	+	***	−	***	**875**
**cellular carbohydrate metabolic process**	+	***			**102**
**primary metabolic process**	+	***	−	***	**1409**
**carbohydrate metabolic process**	+	***			**228**
nucleobase, nucleoside, nucleotide and nucl. acid m. proc.			−	***	696
**cellular amino acid and derivative metabolic process**	+	***	−	*	**113**
**protein metabolic process**	+	***	−	***	**378**
**small molecule metabolic process**	+	***	−	*	**349**
**alcohol metabolic process**	+	**			**64**
**organic acid metabolic process**	+	***			**139**
**cellular amino acid and derivative metabolic process**	+	***	−	*	**113**
**cellular ketone metabolic process**	+	***	−	**	**139**
immune system process			+	**	27
immune response			+	***	26
antigen processing and presentation			*+*	**	*4*
**viral reproduction**					**123**
viral reproductive process					
viral assembly, maturation, egress, and release			+	*	25
virion assembly			+	*	20
viral capsid assembly			*+*	*	*10*
**cellular process**	+	***	−	***	**1782**
cell communication	−	*			**35**
**cellular metabolic process**	+	***	−	***	**1383**
regulation of cellular process			−	**	332

Table 5. Cont.

GO Categories	SRV		PS		#pfam
	Over(+)/ Under(−) represen.	Signif.	Over(+)/ Under(−) represent.	Signif.	
cellular localization			−	***	85
developmental process			+	*	89
response to stimulus			+	**	202
response to stress			+	*	132
defense response	−	**	+	***	**36**
response to wounding			+	**	*9*
immune response			+	***	26
**localization**	+	***	−	**	**360**
macromolecule localization			−	**	104
**establishment of localization**	+	***	−	**	**344**
cellular localization			−	***	85
multi-organism process	−	**	+	***	**142**
pathogenesis			+	**	71
biological regulation			−	**	384
regulation of biological process			−	**	356
regulation of metabolic process			−	**	260
regulation of cellular process			−	**	332

Notation: Significance levels are at the 5% (*), 1% (**), or 0.1% (***). Boldface indicates overrepresentation of SRV; italics indicates underrepresentation of SRV.
doi:10.1371/journal.pone.0095034.t005

a gene is annotated for all the functions and processes of its products, so it may happen that positive selection in a gene is due to positive selection only in a certain protein domain while the signal for positive selection will be tracked for all the functions and processes that the gene is annotated, i.e. all the pathway annotations of its products.

Further, we found an overrepresentation of SRV among the genes participating in some genetic and environmental information processing pathways. We observed underrepresentation of PS among the genes involved in genetic information processing pathways, but overrepresentation of PS among the genes involved in environmental information processing pathways.

Among the cellular processes, cell motility and communication, endocrine and sensory system, and developmental pathways were found to be overrepresented with SRV+ genes. Categories overrepresented with PS+ genes included cell communication and immune and sensory system pathways. These findings are generally consistent with our previous findings for SRV in cellular processes using GO annotations. However, note that the hierarchical structure of cellular processes in KEGG and GO databases is different. For example, GO terms for immune system processes are not "descendants" of terms for cellular processes, while in KEGG cellular process pathways include immune, nervous and sensory system pathways. Therefore, a simple comparison of trends for cellular processes in KEGG and GO is not possible without looking into the finer sub-categories. If the immune, nervous and sensory system pathways were excluded from the KEGG cellular process pathways, then overrepresentation of PS+ in the cellular processes group could not be observed.

Generally, significant overrepresentation of SRV was found among genes involved in human diseases. SRV+ genes were enriched in cancer related pathways (Figure 2). Very strong overrepresentation of SRV+ genes was also found in metabolic disorders (type II diabetes mellitus) and immune disorders (systemic lupus erythematosus). Underrepresentation of genes with SRV was detected among genes involved in neurodegenerative disease pathways. Immune and metabolic disorders pathways exhibited an overrepresentation of PS+ genes.

Site-to-site SRV and Gene Expression Patterns

To test if SRV+ genes are over/underrepresented among the genes expressed in different human tissues, we analyzed gene expression data of 8,175 human genes from HumanProteinpedia (HPRD) expressed in 57 healthy and 20 disease tissues, which were uniquely mapped to KEGG genes. Significant evidence of overrepresentation of SRV+ genes was found among genes expressed in brain, cerebrospinal fluid, liver and pancreatic juice. Among the genes expressed in blood plasma there was an overrepresentation of PS+ genes, while conserved genes were overrepresented among the genes expressed in brain, ovary and stem cell. Indeed, in a previous study genes expressed in the brain were among the most conserved genes with the least evidence for PS [61]. Note that in that study blood plasma was not analyzed as a separate tissue.

Further, we tested for possible relation between gene expression breadth, measured by the number of expression tissues, and the SRV/PS forces. Several studies report that broadly expressed genes evolve more slowly than tissue specific genes (eg. [63,64]). The power for detecting such correlation is very limited with the HPRD data, as it is skewed towards low expression breaths (Figure S5). Therefore, to analyze the correlation between gene expression breadth and SRV/PS we used data from [51] that mapped Ensembl gene IDs to gene expression breadth values estimated from Gene Atlas microarray, EST and SAGE experiments for

Table 6. Over/under-representation of selective forces in KEGG Pathways.

KEGG Pathway	*SRV*			PS		
	Over(+)/ Under(−) represen.	Sign.	#Genes	Over(+)/ Under(−) represen.	Sign.	#Genes
Metabolism			1434	−	**	1484
**Carbohydrate Metabolism**	+	*	300			313
**Pentose phosphate pathway**	+	**	26			26
Pentose and glucuronate interconversions			25	+	***	25
**Fructose and mannose metabolism**	+	*	34			36
Ascorbate and aldarate metabolism			26	+	***	26
**Starch and sucrose metabolism**	+	**	54	+	***	54
**Inositol phosphate metabolism**	+	**	51			51
Energy Metabolism	−	***	170	−	***	178
Oxidative phosphorylation	−	***	116			124
**Nitrogen metabolism**	+	*	24			24
Lipid Metabolism			317			330
**Androgen and estrogen metabolism**	+	*	44	+	***	46
alpha-Linolenic acid metabolism	−	**	17	+	*	17
**Amino Acid Metabolism**	+	*	295			303
**Glycine, serine and threonine metabolism**	+	**	41			41
Glycan Biosynthesis and Metabolism	−	***	206			213
Glycosaminoglycan degradation			18	+	*	18
Glycosphingolipid biosynthesis - globoseries	−	***	14			14
Glycosphingolipid biosynthesis - ganglioseries	−	***	21			21
**Metabolism of Cofactors and Vitamins**	+	*	190			204
**Retinol metabolism**	+	*	56	+	***	65
Porphyrin and chlorophyll metabolism			41	+	***	41
Xenobiotics Biodegradation and Metabolism			156	+	***	160
**Metabolism of xenobiotics by cytochrome P450**	+	***	66	+	***	70
**Drug metabolism - cytochrome P450**	+	***	68	+	***	72
Drug metabolism - other enzymes			52	+	***	52
**Genetic Information Processing**	+	*	560	−	***	573
Translation			143			143
**Aminoacyl-tRNA biosynthesis**	+	**	40			40
**Folding, Sorting and Degradation**	+	*	257			264
**Ubiquitin mediated proteolysis**	+	*	125	−	***	132
**SNARE interactions in vesicular transport**	+	*	37			37
Regulation of autophagy	−	**	34	+	***	34
**Environmental Information Processing**	+	***	1434	+	***	1480
**Membrane Transport**	+	***	42			42
**ABC transporters**	+	***	42			42
**Signal Transduction**	+	***	849			892
**MAPK signaling pathway**	+	***	265			272
**ErbB signaling pathway**	+	***	85			85
**Calcium signaling pathway**	+	***	170			181
**Phosphatidylinositol signaling system**	+	***	69			75
**Hedgehog signaling pathway**	+	*	56			57
Jak-STAT signaling pathway	−	***	126			145
**Signaling Molecules and Interaction**	+	***	729	+	***	750
**Neuroactive ligand-receptor interaction**	+	**	292	+	***	295
**ECM-receptor interaction**	+	*	84	+	***	84

Table 6. Cont.

KEGG Pathway	*SRV*			**PS**		
	**Over(+)/ Under(−) represen.**	**Sign.**	**#Genes**	**Over(+)/ Under(−) represen.**	**Sign.**	**#Genes**
**Cell adhesion molecules (CAMs)**	+	***	**128**	+	***	130
**Cellular Processes**	+	***	**1774**	+	***	1837
**Cell Motility**	+	***	**201**			213
**Regulation of actin cytoskeleton**	+	***	**201**			213
Cell Growth and Death			210	−	*	225
**Cell Communication**	+	***	**400**	+	***	413
**Focal adhesion**	+	***	**193**			201
**Adherens junction**	+	**	**77**			78
**Tight junction**	+	***	**116**	+	***	128
**Gap junction**	+	***	**96**			96
**Endocrine System**	+	*	**369**			381
**Insulin signaling pathway**	+	*	**129**			136
**Melanogenesis**	+	***	**96**			102
Adipocytokine signaling pathway			61	−	*	66
Immune System			519	+	***	547
Antigen processing and presentation	−	*	**82**	+	***	86
Natural killer cell mediated cytotoxicity			132	+	***	139
**Leukocyte transendothelial migration**	+	***	**109**	+	***	117
**Sensory System**	+	***	**416**	+	***	429
**Olfactory transduction**	+	***	**370**	+	***	381
**Taste transduction**	+	*	**51**	+	***	53
**Development**	+	***	**124**			129
**Axon guidance**	+	***	**124**			129
**Human Diseases**	+	***	**983**			1025
**Cancers**	+	***	**365**			378
**Pathways in cancer**	+	***	**305**			312
**Colorectal cancer**	+	***	**83**			83
**Endometrial cancer**	+	**	**50**			50
**Basal cell carcinoma**	+	***	**54**			55
**Melanoma**	+	***	**68**			69
**Immune Disorders**	+	**	**225**	+	*	229
Asthma			30	+	***	30
Autoimmune thyroid disease			53	+	***	53
**Systemic lupus erythematosus**	+	***	**143**			143
Allograft rejection			38	+	***	38
Graft-versus-host disease			42	+	***	42
Neurodegenerative Diseases			275			297
Alzheimer's disease	−	**	**145**			162
Parkinson's disease	−	***	**116**			124
Huntington's disease	−	**	**162**			172
**Metabolic Disorders**	+	***	**96**	+	**	104
**Type II diabetes mellitus**	+	***	**42**			43
Type I diabetes mellitus			42	+	***	44
**Infectious Diseases**	+	***	**147**			149
**Pathogenic Escherichia coli infection**	+	**	**53**			53

Notation: Significance levels are at the 5% (*), 1% (**), or 0.1% (***). Boldface indicates overrepresentation of SRV; italics indicates underrepresentation of SRV.
doi:10.1371/journal.pone.0095034.t006

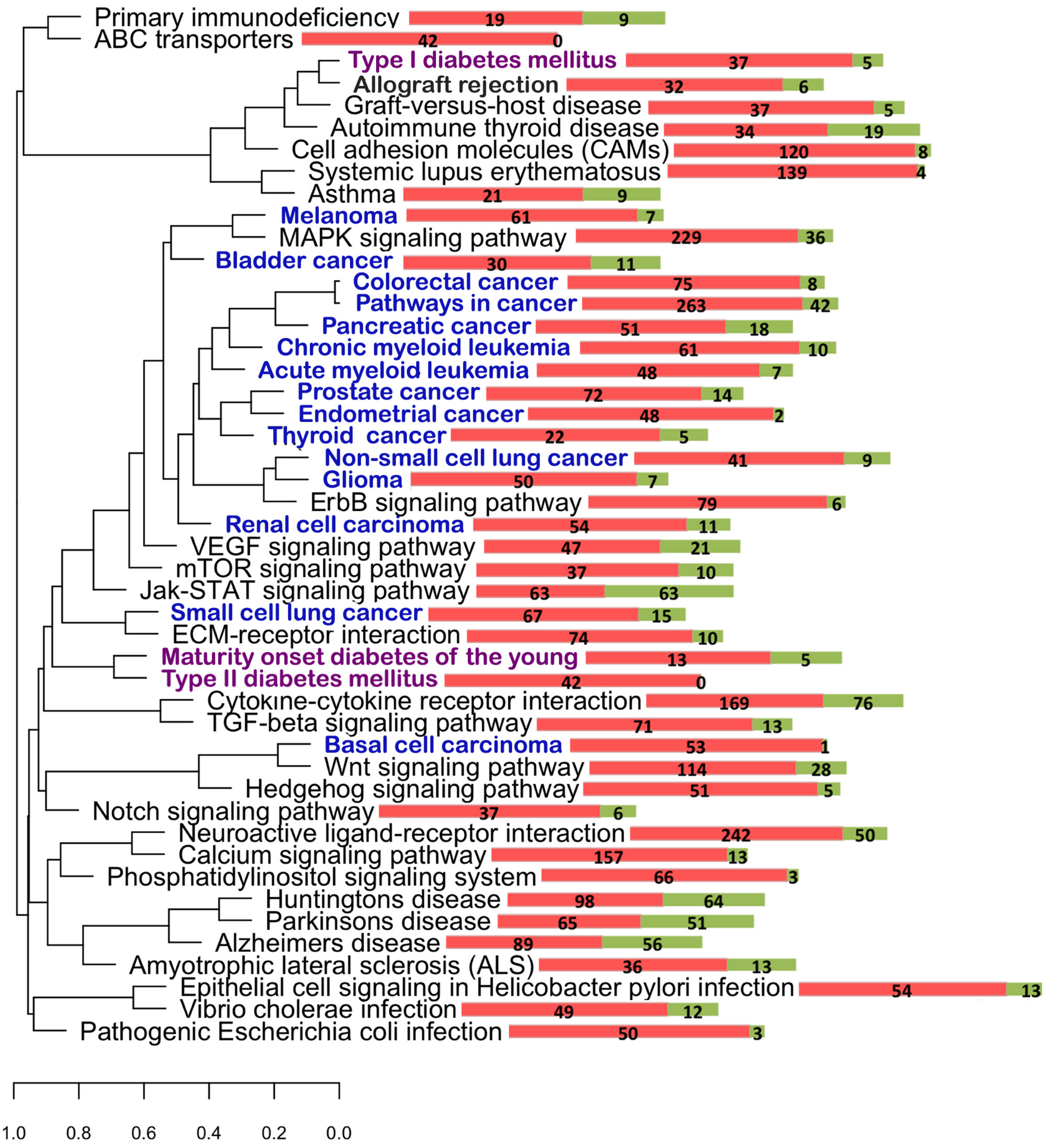

Figure 2. Hierarchical clustering of human disease and environmental information processing pathways in respect to the SRV+ genes that are shared between the pathways. The bars next to the pathways denote the number of SRV+ genes (red) and SRV- genes (green) in the corresponding pathways. Cancer related pathways are marked in blue; metabolic disease pathways are in purple. Note that ABC transporters and Type II diabetes mellitus pathways are exclusively composed of SRV+ genes.
doi:10.1371/journal.pone.0095034.g002

human tissues. Our analyses revealed negative correlation between expression breadth and the average CV of d_S ($\rho = -0.81$, $P = 0.02$) and the average ω-ratio ($\rho = -0.79$, $P = 0.02$) using Gene Atlas microarray data (Figure S6). Similar results were obtained using SAGE and EST data (Figure S7–S8).

Additionally, we used expression measurements in 86 tissues from Gene Atlas Affymetrix U133A microarray. 4,095 proteins that were classified into SRV+/− and PS+/− groups were mapped to the microarray probes. We examined mRNA expression levels of SRV+ and SRV− genes and observed no difference. However, genes with extreme SRV (CV $\geq$ 0.8), showed increased expression levels in nearly all tissues. There were 243 such genes and we refer to them as SRV_{EXT} genes. We compared the distribution of the expression levels of the SRV_{EXT} gene group to the distribution of the expression levels of the SRV− genes. The differences were the most pronounced in several neural tissues: hypothalamus, medulla oblongata, occipital lobe, pineal day, pineal night, prefrontal cortex, spinal cord, amygdala, caudate nucleus, cingulate cortex, fetal brain, whole brain. Figure 3A shows the differences in the distribution of the

expression levels in several tissues. The observed differences remained when we compared the distribution of the expression levels of the SRV_{EXT} genes to the distribution of the expression levels of the SRV− genes including the subgroup of SRV+ genes where CV of d_S was <0.8. Consistently with the study of Kosiol et al. [61], we observed decreased expression levels of PS+ genes in all tissues (Figure 3B). Using this gene expression dataset we observed significant overrepresentation of SRV among the genes expressed in small intestine, pancreas, tongue and several brain tissues. With the Gene Atlas Affymetrix microarray data cerebrospinal fluid and pancreatic juice were not experimentally tested as separate tissues.

Discussion

Large-scale scans for adaptively evolving genes have provided valuable insights into the patterns of positive selection in protein-coding genes, but have left many important questions unanswered. In coding sequences selection may also operate on synonymous sites, contributing to significant variability patterns with respect to the conservation of the synonymous substitution rate and codon usage.

Our analyses of protein families and domains revealed that the site-to-site SRV is a ubiquitous phenomenon affecting over a third of homologous protein domains and families. Strikingly, our study suggests that variation in synonymous rates is more likely in genes that are conserved and are least likely to undergo adaptation at the protein level. Proteins with significant SRV are involved in complex functions, exhibit stronger codon bias and tRNA reusage, have larger number of interactions and participate in forming a larger number of structural complexes. In contrast, we found that genes affected by positive selection tend to have weaker codon bias and fewer interaction partners and form fewer protein complexes. This is consistent with the previous findings: several studies found that the connectivity of proteins in the network is negatively correlated with their rate of evolution [54,65,66].

It has been suggested that proteins with more interactions evolve more slowly because different interactions typically depend on different sites, and so a greater part of the protein is under strong functional constraint [65]. At sites important for interaction between proteins, evolutionary changes may occur largely by co-evolution, in which substitutions in one protein result in selection pressure for reciprocal changes in interacting partners. While we found weak negative correlation between the strength of positive selection and the number of structural complexes, this was not found significant in [54], most likely because at that time the number of structural complexes in Pfam was underestimated (with fewer structures known) and due to smaller size of PANDIT. However, it was shown that families and protein domains that form at least one structure tend to be more conserved. This could suggest that selection acts on all members of the complex, irrespective of the number of complexes formed by each member of the complex [54].

Another surprising finding of our study is that positive selection on the protein tends to be in an antagonistic relationship with forces responsible for the SRV − a trend seen in most of our analyses of gene features (codon/tRNA bias, expression, function). For example, protein domains (very stable protein units optimized

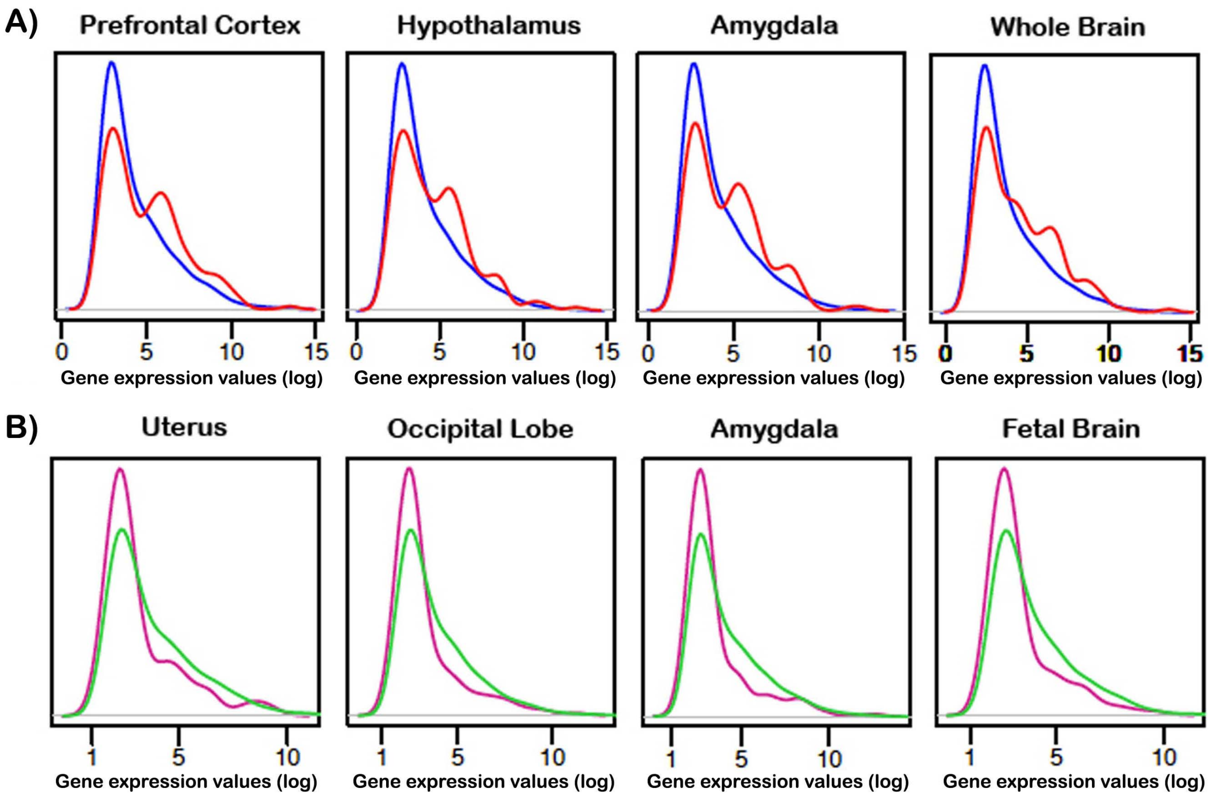

Figure 3. Distribution of the expression levels in A) SRV− genes (blue) and SRV_{EXT} genes (red) and B) PS− genes (green) and PS+ genes (purple) for different tissues. SRV_{EXT} genes show higher expression levels compared to SRV− genes; PS+ genes show reduced expression levels compared to PS− genes.

doi:10.1371/journal.pone.0095034.g003

through deep evolutionary times) evolve slowly compared to protein families (which often evolve under changing evolutionary constraints after gene duplications). Here we found that domains were less likely to undergo positive selection on the protein, but more likely to have SRV. Possibly for domains, protein "building blocks" that are reused in different protein architectures, the exploration of the synonymous mutational landscape is the best way of fine-tuning the already well-optimized amino acid sequence. In contrast, protein families were found to be more likely to undergo positive selection on the protein, but less likely to have significant SRV. Gene ontologies enriched with SRV were often underrepresented with PS.

This may suggest that site-to-site variation of synonymous rates and codon bias are more likely to produce more subtle effects on protein transcription and translation, and so the SRV might be one of the mechanisms of adaptation in the proteins that evolve slowly. Indeed, in very conserved proteins most (if not all) nonsynonymous mutations would result in a dysfunctional protein product and would be selected against. The exploration of mutational landscape is then possible mostly through synonymous mutations. For example, depending on the position in a sequence the use of rare (or optimal) codons may slow down (or speed up) the translation, which can be crucial for correct protein folding [67]. In another example, differences in mRNA stability were attributed to synonymous mutations in the conserved gene *lady bird early* (lbe) from the homeobox cluster of *Drosophila melanogaster* [68]. This example is consistent with our observations: on lbe balancing selection on synonymous sites acts at the background of strict purifying selection on the protein.

Crucially, the understanding of protein function requires a detailed analysis of sequence-structure-function trinity. Here we focused on sequences with SRV, a phenomenon that may affect protein folding, abundance, degradation and function - through the regulation of translational rate or mRNA stability. In our study, proteins found in the cell interior (with exception of organelles) tended to have more SRV, while it was observed less frequently in proteins located in the extracellular region. Again, this pattern is opposite to the well-known localization pattern for proteins whose protein encoding sequences are under positive selection on the protein level. Furthermore, proteins involved in metabolic and cellular processes, transporter activities and binding exhibited significant excess of SRV.

Several pathways are especially rich in genes with SRV, suggesting that selective forces on synonymous sites may frequently act directly on whole protein complexes or pathways. This can be seen from our clustering of SRV genes by KEGG terms, where several disease pathways and related environmental information processing pathways frequently share many genes with SRV (Figure 2). This is supported by recent literature reporting known associations of synonymous mutations with >40 human diseases [24].

Alternatively, some studies suggested that adaptive changes in one protein may sometimes have a cascade effect, leading to changes in other genes that bring a system back into the equilibrium [69]. Further investigation in this respect is needed in order to analyze the effects of the synonymous changes along the pathway and to reveal the reasons for overrepresentation of genes with SRV in certain pathways.

Genes expressed in certain tissues (brain, cerebrospinal fluid, liver, pancreatic juice) showed excess of SRV. Moreover, genes with extreme SRV had increased expression levels in most of the human tissues, especially in brain tissues. This may indicate that codon bias towards optimal codons, which correlates with gene expression, may not affect all sites, but is often a site-specific phenomenon. Indeed, as mentioned above, variation in usage of optimal vs rare codons could act as a mechanism for regulating the speed of translation along the sequence, consistent with the co-translational folding hypothesis. Some recent studies suggested that site-specific codon preferences may be better explained by pressures for translational accuracy [70–72] rather than speed of translation, and the impact of rare codon clusters on ribosomal occupancy has been recently questioned based on ribosomal footprinting in yeast [73]. This highlights the complexity of the relationship between selection on synonymous sites, biochemical properties of the transcript, protein production and the eventual function of protein product, necessitating further studies in this direction.

Recent reports show that synonymous SNPs (synSNPs) can be associated with disease phenotype, causing disease or be responsible for differences in individual responses to drug treatment. If a haplotype with a synSNP has higher fitness, it will increase in frequency due to selection. Growing number of diseases are associated with synonymous polymorphisms, such as several types of cancers, hyperinsulinism of infancy, diabetes, and prion-related conditions, to name a few [24,67,74,75,76,77,78]. Indeed, in our data we observed high SRV in genes associated with diabetes, lupus and various cancers. We found significant SRV in several human genes where synSNPs have been documented to lead or contribute to a disease [25], among such examples are: the CHRNE gene, where a synSNP can directly cause a Myastenic syndrome (muscle disease); the FGFR2 gene, where a synSNP is a direct cause of a Crouzon syndrome (bone disease); the tumor suppressor protein p53, where synonymous polymorphisms are associated with overall tumor susceptibility, pathology and prognosis; the EGFR gene, where synSNPs may be a potential predictor for clinical outcome in advanced Non-Small-Cell Lung carcinoma; the PAH gene, where synSNPs can lead to Phenylketonuria; the CHRNA4 gene, where synSNPs are associated with Alzheimer's disease; in the three genes PADI2, SYNGR1 and DRD2 associated with schizophrenia. Interestingly, we also detected significant SRV in the MDR-1 gene − the first known case where the effect of a synonymous change on protein folding was demonstrated in vivo (discussed in the introduction; [21,79]). Our analyses identified overrepresentation of SRV in metabolizing enzymes and transporters, which are subject to many pharmacogenetics studies because they determine the disposition, safety and efficacy of small molecule drugs [24].

Overall, the SRV statistic carries a real signal, identifying important genes including those associated to human disease. However, like for any automated large-scale study, the conclusions should not be overgeneralized and taken with caution: hidden effects such as errors in annotation and reduced power of LRTs for small or too divergent alignments may have contributed to the overall signal (indeed in our data correlation was found with number of taxa and divergence, although weak and clearly non-linear (see Figure S9)). The possibility that size/divergence of alignments may cause variation in power of LRT for positive selection (which are methodologically quite similar to the LRT for SRV that we used here) has been thoroughly studied in [38] using computer simulations. The study showed that for small alignments and too low/deep divergences the LRT remained accurate but had decreased power. To check that this did not bias our results, we repeated all analyzes by removing small alignments (in different combinations) with and without a threshold of ≥0.3 on the CV of SRV. We could confirm the reported trends in all cases.

Whole-genome investigations on a fixed number of lineages would help to reduce some of the above-mentioned effects. Further, to detect positions affected by site-specific selection on synonymous changes with sufficient confidence, better models and tests need to be developed, taking into account site-to-site codon variability. Better understanding of site-specific synonymous variability promises to become an important contribution to revising the central molecular biology concepts, to improving structural prediction, and to our understanding of genetic diseases with respect to potential effects of synonymous mutations.

Supporting Information

Figure S1 Histogram of PANDIT data sets divergence. The divergence (expected substitutions per amino acid site per branch) was calculated as AA tree length divided by 2*T-3, where T is the number of sequences in the PANDIT data set. The AA tree length and the number of sequences in the each data set were extracted from PANDIT.
(TIF)

Figure S2 Bootstrap distributions of the differences in mean values of A) Codon Bias Indices (CBI) and B) Effective Number of Codons (ENC) between protein groups showing evidence for site-to-site variation in synonymous rates (SRV+) and those failing to show such evidence (SRV−), and protein groups showing evidence for positive selection (PS+) and those failing to show such evidence (PS−). The differences are significant, since 95% of the histogram area does not include the zero value for all histograms.
(TIF)

Figure S3 Bootstrap distributions of the differences in A) mean GC content values and B) GC3 content values between PANDIT members showing evidence for site-to-site variation in synonymous rates (SRV+) and those failing to show such evidence (SRV−), and PANDIT members showing evidence for positive selection (PS+) and those failing to show such evidence (PS−). All the differences are significant, since 95% of the histogram area does not include the zero value for all histograms.
(TIF)

Figure S4 Distributions of data in GO terms.
(TIF)

Figure S5 Expression breadth histogram of genes in HumanProteinpedia Database.
(TIF)

Figure S6 Correlation between gene expression breadth (number of tissues of gene expression) calculated from human Gene Atlas microarray data and A) average CV of synonymous rates and B) average ω ratio, calculated for each bin of 10 tissues. The Gene Atlas microarray expression breadth values were taken from Necsulea et al. (2009).
(TIF)

Figure S7 Correlation between gene expression breadth (number of tissues of gene expression) calculated from human SAGE data and A) average CV of synonymous rates and B) average ω ratio. The SAGE gene expression breadth values were taken from Necsulea et al. (2009).
(TIF)

Figure S8 Correlation between gene expression breadth (number of tissues of gene expression) calculated from human EST data and A) average CV of synonymous rates and B) average ω ratio. The EST gene expression breadth values were taken from Necsulea et al. (2009).
(TIF)

Figure S9 Correlation between individual variables (stated in the diagonal). The numbers in the upper-diagonal plots denote the correlation coefficients for the corresponding pairs of variables. The lower-diagonal plots represent plots of the corresponding data.
(TIF)

Table S1 PFAM protein groups with extreme site-to-site heterogeneity of synonymous rates (coefficient of variation (CV) ≥1).
(XLS)

Table S2 Clans that are exclusively composed of PFAM groups identified as having site-to-site heterogeneity of synonymous rates (SRV+).
(XLS)

Table S3 Strength of correlation between codon bias, tRNA reusage and nucleotide composition and SRV/PS. Note that negative correlation with ENC indicates positive correlation to codon bias, since, unlike CBI, smaller ENC indicates stronger codon bias.
(XLS)

Author Contributions

Conceived and designed the experiments: SD MA. Performed the experiments: SD MA. Analyzed the data: SD MA. Contributed reagents/materials/analysis tools: SD. Wrote the paper: SD MA.

References

1. Anfinsen CB (1973) Principles that govern the folding of protein chains. Science 181: 223–230.
2. Clarke B (1970) Darwinian evolution of proteins. Science 168: 1009–1011.
3. Ikemura T (1981) Correlation between the abundance of Escherichia coli transfer RNAs and the occurrence of the respective codons in its protein genes. J Mol Biol 146: 1–21.
4. Akashi H, Eyre-Walker A (1998) Translational selection and molecular evolution. Curr Opin Genet Dev 8: 688–693.
5. Duret L (2002) Evolution of synonymous codon usage in metazoans. Curr Opin Genet Dev 12: 640–649.
6. Wright SI, Yau CB, Looseley M, Meyers BC (2004) Effects of gene expression on molecular evolution in Arabidopsis thaliana and Arabidopsis lyrata. Mol Biol Evol 21: 1719–1726.
7. Gingold H, Pilpel Y (2011) Determinants of translation efficiency and accuracy. Mol Syst Biol 7: 481.
8. Frenkel-Morgenstern M, Danon T, Christian T, Igarashi T, Cohen L, et al. (2012) Genes adopt non-optimal codon usage to generate cell cycle-dependent oscillations in protein levels. Mol Syst Biol 8: 572.
9. Chamary JV, Parmley JL, Hurst LD (2006) Hearing silence: non-neutral evolution at synonymous sites in mammals. Nat Rev Genet 7: 98–108.
10. Fairbrother WG, Yeh RF, Sharp PA, Burge CB (2002) Predictive identification of exonic splicing enhancers in human genes. Science 297: 1007–1013.
11. Wang Z, Rolish ME, Yeo G, Tung V, Mawson M, et al. (2004) Systematic identification and analysis of exonic splicing silencers. Cell 119: 831–845.
12. Eskesen ST, Eskesen FN, Ruvinsky A (2004) Natural selection affects frequencies of AG and GT dinucleotides at the 5′ and 3′ ends of exons. Genetics 167: 543–550.
13. Hurst LD, Pal C (2001) Evidence for purifying selection acting on silent sites in BRCA1. Trends Genet 17: 62–65.
14. Orban TI, Olah E (2001) Purifying selection on silent sites – a constraint from splicing regulation? Trends Genet 17: 252–253.

15. Iida K, Akashi H (2000) A test of translational selection at 'silent' sites in the human genome: base composition comparisons in alternatively spliced genes. Gene 261: 93–105.
16. Lu H, Lin L, Sato S, Xing Y, Lee CJ (2009) Predicting functional alternative splicing by measuring RNA selection pressure from multigenome alignments. PLoS Comput Biol 5: e1000608.
17. Xing Y, Lee C (2006) Alternative splicing and RNA selection pressure–evolutionary consequences for eukaryotic genomes. Nat Rev Genet 7: 499–509.
18. Duan J, Wainwright MS, Comeron JM, Saitou N, Sanders AR, et al. (2003) Synonymous mutations in the human dopamine receptor D2 (DRD2) affect mRNA stability and synthesis of the receptor. Hum Mol Genet 12: 205–216.
19. Nackley AG, Shabalina SA, Tchivileva IE, Satterfield K, Korchynskyi O, et al. (2006) Human catechol-O-methyltransferase haplotypes modulate protein expression by altering mRNA secondary structure. Science 314: 1930–1933.
20. Capon F, Allen MH, Ameen M, Burden AD, Tillman D, et al. (2004) A synonymous SNP of the corneodesmosin gene leads to increased mRNA stability and demonstrates association with psoriasis across diverse ethnic groups. Hum Mol Genet 13: 2361–2368.
21. Kimchi-Sarfaty C, Oh JM, Kim IW, Sauna ZE, Calcagno AM, et al. (2007) A "silent" polymorphism in the MDR1 gene changes substrate specificity. Science 315: 525–528.
22. Thanaraj TA, Argos P (1996) Ribosome-mediated translational pause and protein domain organization. Protein Sci 5: 1594–1612.
23. Oresic M, Shalloway D (1998) Specific correlations between relative synonymous codon usage and protein secondary structure. J Mol Biol 281: 31–48.
24. Sauna ZE, Kimchi-Sarfaty C, Ambudkar SV, Gottesman MM (2007) Silent polymorphisms speak: how they affect pharmacogenomics and the treatment of cancer. Cancer Res 67: 9609–9612.
25. Sauna ZE, Kimchi-Sarfaty C (2011) Understanding the contribution of synonymous mutations to human disease. Nat Rev Genet 12: 683–691.
26. Chamary JV, Hurst LD (2009) The price of silent mutations. Sci Am 300: 46–53.
27. Chen R, Davydov EV, Sirota M, Butte AJ (2010) Non-synonymous and synonymous coding SNPs show similar likelihood and effect size of human disease association. PLoS One 5: e13574.
28. Anisimova M, Liberles D (2012) Detecting and understanding natural selection. In: Cannarozzi G, Schneider A, editors. Codon Evolution: mechanisms and models: Oxford University Press.
29. Resch AM, Carmel L, Marino-Ramirez L, Ogurtsov AY, Shabalina SA, et al. (2007) Widespread positive selection in synonymous sites of mammalian genes. Mol Biol Evol 24: 1821–1831.
30. Yang Z, Bielawski JP (2000) Statistical methods for detecting molecular adaptation. Trends Ecol Evol 15: 496–503.
31. Zhou T, Gu WJ, Wilke CO (2010) Detecting Positive and Purifying Selection at Synonymous Sites in Yeast and Worm. Molecular Biology and Evolution 27: 1912–1922.
32. Whelan S, de Bakker PI, Quevillon E, Rodriguez N, Goldman N (2006) PANDIT: an evolution-centric database of protein and associated nucleotide domains with inferred trees. Nucleic Acids Res 34: D327–331.
33. Punta M, Coggill PC, Eberhardt RY, Mistry J, Tate J, et al. (2012) The Pfam protein families database. Nucleic Acids Res 40: D290–301.
34. Guindon S, Dufayard JF, Lefort V, Anisimova M, Hordijk W, et al. (2010) New algorithms and methods to estimate maximum-likelihood phylogenies: assessing the performance of PhyML 3.0. Syst Biol 59: 307–321.
35. Dimitrieva S, Anisimova M (2010) PANDITplus: toward better integration of evolutionary view on molecular sequences with supplementary bioinformatics resources. Trends in Evol Biol 2.
36. Yang Z (2007) PAML 4: phylogenetic analysis by maximum likelihood. Mol Biol Evol 24: 1586–1591.
37. Yang Z, Nielsen R, Goldman N, Pedersen AM (2000) Codon-substitution models for heterogeneous selection pressure at amino acid sites. Genetics 155: 431–449.
38. Anisimova M, Bielawski JP, Yang Z (2001) Accuracy and power of the likelihood ratio test in detecting adaptive molecular evolution. Molecular biology and evolution 18: 1585–1592.
39. Anisimova M, Bielawski JP, Yang Z (2002) Accuracy and power of bayes prediction of amino acid sites under positive selection. Molecular biology and evolution 19: 950–958.
40. Wong WSW, Yang Z, Goldman N, Nielsen R (2004) Accuracy and power of statistical methods for detecting adaptive evolution in protein coding sequences and for identifying positively selected sites. Genetics 168: 1041–1051.
41. Pond SLK, Frost SDW, Muse SV (2005) HyPhy: hypothesis testing using phylogenies. Bioinformatics (Oxford, England) 21: 676–679.
42. Benjamini Y, Hochberg Y (1995) Controlling the false discovery rate: a practical and powerful approach to multiple testing. J R Statist Soc B 57: 289–300.
43. Bennetzen JL, Hall BD (1982) Codon Selection in Yeast. Journal of Biological Chemistry 257: 3026–3031.
44. Wright F (1990) The Effective Number of Codons Used in a Gene. Gene 87: 23–29.
45. Peden J (1999) CodonW. PhD Thesis, University of Nottingham.
46. Friberg MT, Gonnet P, Barral Y, Schraudolph NN, Gonnet GH (2006) Measures of codon bias in yeast, the tRNA Pairing Index and possible DNA repair mechanisms. Algorithms in Bioinformatics, Proceedings 4175: 1–11.
47. Cannarozzi G, Schraudolph NN, Faty M, von Rohr P, Friberg MT, et al. (2010) A role for codon order in translation dynamics. Cell 141: 355–367.
48. Gonnet GH, Hallett MT, Korostensky C, Bernardin L (2000) Darwin v. 2.0: an interpreted computer language for the biosciences. Bioinformatics 16: 101–103.
49. Roth A, Anisimova M, Cannarozzi G (2012) Measuring codon usage bias. In: Cannarozzi G, Schneider A, editors. Codon Evolution: mechanisms and models: Oxford University Press.
50. Kandasamy K, Keerthikumar S, Goel R, Mathivanan S, Patankar N, et al. (2009) Human Proteinpedia: a unified discovery resource for proteomics research. Nucleic Acids Res 37: D773–781.
51. Necsulea A, Semon M, Duret L, Hurst LD (2009) Monoallelic expression and tissue specificity are associated with high crossover rates. Trends Genet 25: 519–522.
52. Flicek P, Amode MR, Barrell D, Beal K, Brent S, et al. (2011) Ensembl 2011. Nucleic Acids Res 39: D800–806.
53. Su AI, Wiltshire T, Batalov S, Lapp H, Ching KA, et al. (2004) A gene atlas of the mouse and human protein-encoding transcriptomes. Proc Natl Acad Sci U S A 101: 6062–6067.
54. Aris-Brosou S (2005) Determinants of adaptive evolution at the molecular level: the extended complexity hypothesis. Mol Biol Evol 22: 200–209.
55. Chamary JV, Hurst LD (2005) Biased codon usage near intron-exon junctions: selection on splicing enhancers, splice-site recognition or something else? Trends Genet 21: 256–259.
56. Eyre-Walker A (1999) Evidence of selection on silent site base composition in mammals: potential implications for the evolution of isochores and junk DNA. Genetics 152: 675–683.
57. Hughes AL, Yeager M (1997) Comparative evolutionary rates of introns and exons in murine rodents. J Mol Evol 45: 125–130.
58. Amit M, Donyo M, Hollander D, Goren A, Kim E, Gelfman S, Lev-Maor G, Burstein D, Schwartz S, Postolsky B, Pupko T, Ast G. Differential GC content between exons and introns establishes distinct strategies of splice-site recognition. Cell Rep. 2012 May 31;1(5): 543–56.
59. Najafabadi HS, Salavati R (2008) Sequence-based prediction of protein-protein interactions by means of codon usage. Genome Biol 9: R87.
60. Ashburner M, Ball CA, Blake JA, Botstein D, Butler H, et al. (2000) Gene ontology: tool for the unification of biology. The Gene Ontology Consortium. Nat Genet 25: 25–29.
61. Kosiol C, Vinar T, da Fonseca RR, Hubisz MJ, Bustamante CD, et al. (2008) Patterns of positive selection in six Mammalian genomes. PLoS Genet 4: e1000144.
62. Nielsen R, Bustamante C, Clark AG, Glanowski S, Sackton TB, et al. (2005) A scan for positively selected genes in the genomes of humans and chimpanzees. PLoS Biol 3: e170.
63. Zhang LQ, Li WH (2004) Mammalian housekeeping genes evolve more slowly than tissue-specific genes. Molecular Biology and Evolution 21: 236–239.
64. Park SG, Choi SS (2010) Expression breadth and expression abundance behave differently in correlations with evolutionary rates. Bmc Evolutionary Biology 10.
65. Fraser HB, Hirsh AE, Steinmetz LM, Scharfe C, Feldman MW (2002) Evolutionary rate in the protein interaction network. Science 296: 750–752.
66. Yook SH, Oltvai ZN, Barabasi AL (2004) Functional and topological characterization of protein interaction networks. Proteomics 4: 928–942.
67. Komar AA (2009) A pause for thought along the co-translational folding pathway. Trends Biochem Sci 34: 16–24.
68. Balakirev ES, Anisimova M, Ayala FJ (2011) Complex Interplay of Evolutionary Forces in the ladybird Homeobox Genes of Drosophila melanogaster. PLoS ONE 6: e22613.
69. Presgraves DC, Stephan W (2007) Pervasive adaptive evolution among interactors of the Drosophila hybrid inviability gene, Nup96. Molecular Biology and Evolution 24: 306–314.
70. Ingolia NT, Lareau LF, Weissman JS (2011) Ribosome profiling of mouse embryonic stem cells reveals the complexity and dynamics of mammalian proteomes. Cell 147: 789–802.
71. Qian W, Yang J-R, Pearson NM, Maclean C, Zhang J (2012) Balanced codon usage optimizes eukaryotic translational efficiency. PLoS Genet 8: e1002603.
72. Li GW, Oh E, Weissman JS (2012) The anti-Shine-Dalgarno sequence drives translational pausing and codon choice in bacteria. Nature 484: 538–541.
73. Charneski CA, Hurst LD (2013) Positively Charged Residues Are the Major Determinants of Ribosomal Velocity. PLoS Biol 11(3): e1001508.
74. Shah JH, Maguire DJ, Munce TB, Cotterill A (2008) Alanine in HI: a silent mutation cries out! Adv Exp Med Biol 614: 145–150.
75. Komar AA (2007) Genetics. SNPs, silent but not invisible. Science 315: 466–467.
76. Komar AA (2007) Silent SNPs: impact on gene function and phenotype. Pharmacogenomics 8: 1075–1080.
77. Sauna ZE, Kimchi-Sarfaty C, Ambudkar SV, Gottesman MM (2007) The sounds of silence: synonymous mutations affect function. Pharmacogenomics 8: 527–532.
78. Kelly AC, Mateus-Pinilla NE, Diffendorfer J, Jewell E, Ruiz MO, et al. (2008) Prion sequence polymorphisms and chronic wasting disease resistance in Illinois white-tailed deer (Odocoileus virginianus). Prion 2: 28–36.
79. Tsai CJ, Sauna ZE, Kimchi-Sarfaty C, Ambudkar SV, Gottesman MM, et al. (2008) Synonymous mutations and ribosome stalling can lead to altered folding pathways and distinct minima. J Mol Biol 383: 281–291.
